# Synthesis of structurally diverse major groove DNA interstrand crosslinks using three different aldehyde precursors

**DOI:** 10.1093/nar/gku328

**Published:** 2014-04-29

**Authors:** Shivam Mukherjee, Angelo Guainazzi, Orlando D. Schärer

**Affiliations:** 1 Department of Chemistry, Stony Brook University, Stony Brook, NY 11794-3400, USA; 2 Department of Pharmacological Sciences, Stony Brook University, Stony Brook, NY 11794-8651, USA

## Abstract

DNA interstrand crosslinks (ICLs) are extremely cytotoxic lesions that block essential cellular processes, such as replication and transcription. Crosslinking agents are widely used in cancer chemotherapy and form an array of structurally diverse ICLs. Despite the clinical success of these agents, resistance of tumors to crosslinking agents, for example, through repair of these lesions by the cellular machinery remains a problem. We have previously reported the synthesis of site-specific ICLs mimicking those formed by nitrogen mustards to facilitate the studies of cellular responses to ICL formation. Here we extend these efforts and report the synthesis of structurally diverse major groove ICLs that induce severe, little or no distortion in the DNA. Our approach employs the incorporation of aldehyde precursors of different lengths into complementary strands and ICL formation using a double reductive amination with a variety of amines. Our studies provide insight into the structure and reactivity parameters of ICL formation by double reductive amination and yield a set of diverse ICLs that will be invaluable for exploring structure–activity relationships in ICL repair.

## INTRODUCTION

DNA interstrand crosslinks (ICLs) are extremely cytotoxic lesions that covalently connect two complementary strands of a DNA duplex. As DNA strand separation is essential to cellular processes, such as transcription and replication, ICLs are highly cytotoxic (1–3). Bifunctional electrophiles, such as cisplatin, nitrogen mustards (NMs) chloro ethyl nitroso ureas and mitomycin C, form ICLs and are a mainstay as frontline chemotherapeutic drugs (4). Despite the clinical success of these drugs, resistance mechanisms including the removal of ICLs from DNA by cellular proteins contributes to the resistance of tumor cells to treatment with crosslinking agents (5). ICLs are also formed by endogenous bifunctional agents, such as malondialdehyde and formaldehyde (6–8), and such agents have likely been the evolutionary drivers for the cellular responses to ICL formation. The importance of these repair pathways is underscored by the existence of the hereditary cancer prone disorder Fanconi anemia (FA) (9,10). Cells from FA patients display exquisite sensitivity to ICL-forming agents and exposure to crosslinking agents serves as the definitive clinical diagnosis for FA.

The repair of ICLs is a complex process and the pathways involved have been subject of intense recent studies (11). These efforts have been critically dependent on the ability to generate site-specific ICL (reviewed in (12)). Breakthroughs in the synthesis of site-specific ICLs have been achieved by a number of laboratories by incorporating ICL precursors into DNA as phosphoramidites by solid phase DNA synthesis. Such approaches made use of the incorporation of nucleosides crosslinked outside of the DNA (7,13–15) and the incorporation of ICL precursors on one or two strands of DNA and the use of specific reaction to induce ICL formation (16–26). These efforts have provided access to structurally diverse ICLs formed in the major and minor grooves, on the Waston–Crick base pairing face or stacked between the two bases. ICLs induce a variety of alterations in DNA duplex structures, due to differences in the attachment sites on the DNA bases and the chemical composition of the crosslink. Recent studies have revealed that the biological responses triggered by ICL can vary depending on how they influence duplex structure (27–32).

Our own studies have been mainly concerned with major groove ICLs (17,22), the site where adducts are formed by the clinically important cisplatin and nitrogen mustards or the environmental pollutant diepoxybutane. The length of the crosslink agent bridging two N7 atoms of dG residues on opposite strands of ICLs formed in the major groove lead to different degrees of distortion in DNA, dictated by length of the ICL linking the two bases (33–36).

**Figure 1.**
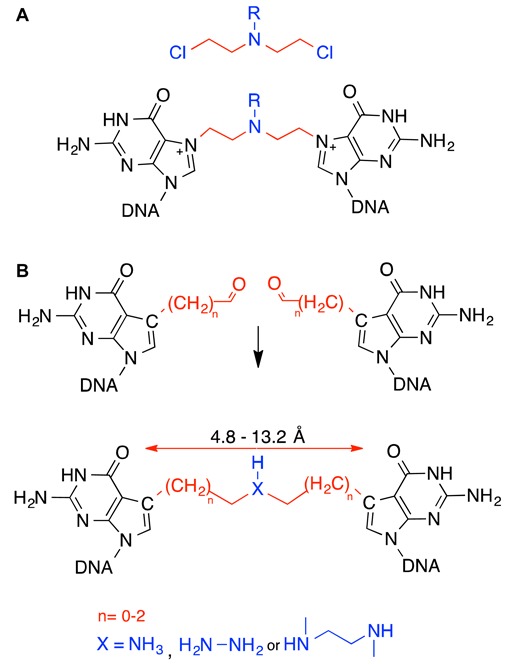
Native and synthetic nitrogen mustard ICLs. **A**. Nitrogen mustard and the DNA ICL formed by reaction with duplex DNA. The structure of the side chain R, varies with the type of nitrogen mustard for example ‘CH_3_’ is for mechlorethamine, the first clinically used antitumor drug. **B**. Our approach to forming ICLs having possible linker lengths from 4.8 to 13.2 Å uses a combination of ICL precursors, a 7-alkylaldehyde-7-deazaguanine with C1, C2 or C3 alkyl chains in complementary strands of a DNA duplex and a double reductive amination reaction with ammonia, hydrazine or DMEDA. Note that guanine residues at the ICL are replaced with 7-deazaguanine to facilitate ICL synthesis and increase stability.

We have synthesized ICLs using precursor molecules containing reactive aldehyde groups masked as diols that were incorporated into complementary strands of a duplex and by using a double reductive amination reaction (Figure 1). Using aldehyde precursors connected to the base through one or two carbon alkyl chains and using a variety of amines, we found that the yield of ICL formation dramatically depended on the length of the ICL formed, and the reactivity of the amine used (17,22). Here we extend these studies through the synthesis of a new ICL precursor and a systematic assessment of reaction conditions for ICL formation, providing access to a variety of NM ICL mimics inducing moderate, mild or no distortion in the DNA duplex. We expect that these structurally diverse ICLs will be invaluable for structure–function relationships of ICL repair, extending previous studies that have started to reveal important differences in how ICLs of different structures are processed in replication-dependent and replication-independent repair (27,29,37).

## MATERIALS AND METHODS

### Synthesis of the C3-aldehyde phosphoramidite 8

The synthesis of phosphoramidite **8**, including protocols for the synthesis and structural characterization (^1^H-NMR, ^13^C-NMR, HR-MS) of each intermediate is described in detail in the Supplementary Data section.

### Oligonucleotide synthesis and purification

Oligonucleotide synthesis was carried out on an Expedite 8909 Nucleic Acid Synthesis System (Applied Biosystems) using 1 μmol 1000 Å CPG –dG and –dC column cartridges (Biosearch Technolgies). An extended coupling time of 15 min was used with all the modified phosphoramidite building blocks. The following sequences were synthesized: 5′-d(GTCACTGGTAG*ACAGCATTG) and 5′-d(CAATGCTG*TCTACCAGTGAC), where G* is the modified phosphoramidite having either C1, C2 or C3 linker. The trityl-on oligonucleotides were deprotected by treatment with concentrated NH_4_OH solution at 50°C for 12 h, then purified and detritylated using 1 μmol TOP-cartridges (Agilent Technologies) following the manufacturer's protocol. The *tert*-butyldimethylsilyl (TBDMS) group in the oligonucleotides containing the C3 aldehyde precursor was removed by treating with TEA·3HF overnight at 40°C followed by precipitation using 1-butanol. Subsequently, oligonucleotides were purified by high pressure liquid chromatography (HPLC) on a C18 column (Phenomenex Clarity 5μ Oligo-RP 50 × 10 mm) using the following elution gradient: linear 2.5–15% B over 21 min, linear 15–90% B over 25 min, isocratic 90% B till 26 min, linear 90–2.5% B till 28 min, isocratic 2.5% B till 30 min (eluent A: 0.1 M TEAA (pH 7); eluent B: CH_3_CN).

### Formation of ICLs by reductive amination

A solution containing two single stranded oligonucleotides (25 nmols in 125 μl 100 mM NaCl) was heated to 95°C and allowed to cool over a period of 5 h for annealing to take place. For oxidation, 10 μl 50 mM NaIO_4_ and 15 μl 1 M sodium phosphate buffer (pH 5.4) was added and mixture was kept at 4°C overnight. Excess NaIO_4_ was removed by centrifugation through Microcon columns with a 3K cutoff (Millipore). The crosslink was formed by adding 10 μl 5 mM aqueous solution of the amine (ammonium acetate, hydrazine or N,N′-dimethylethylene diamine (DMEDA)) and 10 μl of a 0.5 M solution of sodium cyanoborohydride and allowing to react in the dark at room temperature overnight. ICL formation was analyzed by gel electrophoresis on a 20% denaturing polyacrylamide gel (7 M urea). To purify and isolate the ICL-containing oligonucleotide the gel was visualized by UV shadowing and the crosslinked oligonucleotide was excised from the gel and the DNA was extracted by electroelution using the Elutrap device (Schleicher & Schuell). After purification the isolated yields of ICLs were in the range from 5 to 12 nmols.

## RESULTS AND DISCUSSION

### Strategy for the synthesis of structurally diverse ICLs

We have previously reported that NM-like ICLs can be synthesized by incorporation of two aldehyde precursors on complementary strands of DNA and crosslink formation using a double reductive amination reaction (17,22). In the course of these studies, we noted that the efficiency of ICL formation is dependent upon the length of the ICL as well as the reactivity of the amine used in the reaction (Figure 1B). This is illustrated by our attempts to synthesize an ICL isosteric to those formed by NMs that contain a 5-atom chain in the crosslink and induce a bend of about 20° in the DNA (*n* = 1, Figure 1). We observed that formation of this crosslink was unsuccessful with two aldehyde precursors with a C2 alkyl chain and ammonia as the amine, while it could be formed with a C2 and C1 alkyl and hydrazine as an amine. By contrast, an ICL that does not induce a distortion can be formed with a number of different amines (17,22). These examples show that the length of the ICL to be formed and the reactivity of the amine (hydrazine is more nucleophilic than ammonia) determine the success of ICL formation in our approach. We wished to more fully exploit our approach to generate structurally diverse major grove ICLs by double reductive amination and to study the parameters of reactivity that govern crosslink formation.

**Figure 2.**
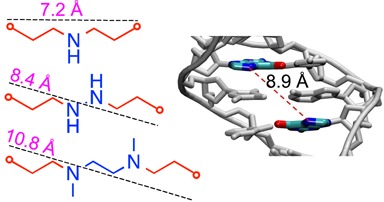
Distance between N7 position on opposing strand of two G residues in B-form DNA and the theoretical length of the alkylamine chain linker in ICLs. The lengths of the alkyl chains are indicated over the dotted lines represent the maximum possible length in a fully extended all *anti*-conformation.

To be able to access ICLs spanning a broad range of distances across the major groove, we set out to synthesize a new ICL precursor with a three-carbon alkylaldehyde (C3) precursor. Together with the previously synthesized C1 and C2 aldehyde precursors and using ammonia, hydrazine and N,N′-dimethylethylenediamine (DMEDA) this should allow us to generate a set of structurally diverse ICLs. We define here the length of an ICL based on the fully extended alkylamine chain spanning the distance between the two crosslinked deazaguanine residues (illustrated in Figure 2). The distance between the atoms at the 7 position of dG where the ICL is attached is approximately 8.9 Å in B-form DNA, so any ICL that spans a length of 8.9 Å or less will induce some form of distortion in the DNA.

### Synthesis of a three-carbon alkyl aldehyde chain precursor

**Figure 3.**
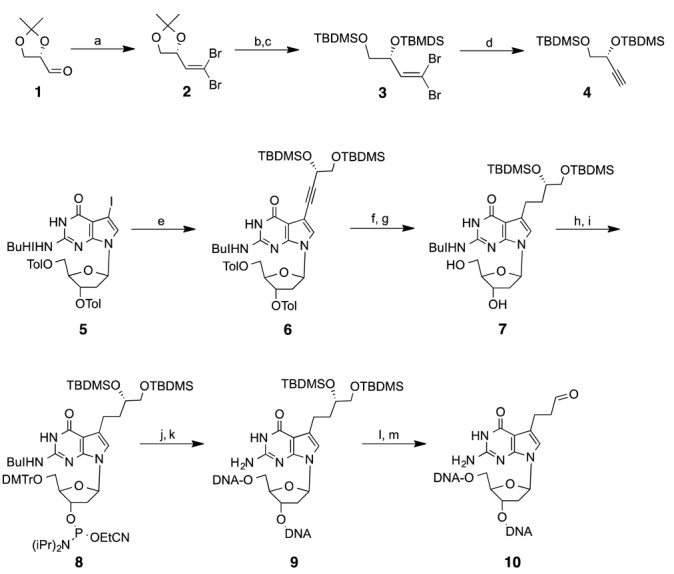
Scheme showing the synthesis of the C3 alkyl chain ICL precursor phosphoramidite **8**. Reagents, conditions and yields: (a) PPh_3_, CBr_4_, CH_2_Cl_2_, 3 h, 84%; (b) Dowex-50W, MeOH, 12 h, 50°C, 95%; (c) TBDMSCl, Imidazole, DMF, 12 h, 83%; (d) EtMgBr, THF, 3 h, 90%; (e) **4**, Pd(PPh_3_)_4_, CuI, Et_3_N, DMF, 24 h, 60°C, 59%; (f) H_2_, Pd/C, EtOAc/MeOH, 42h, 84%; (g) NaOMe, THF, 6 h, 90%; (h) DMTrCl, Py, 1.5 h, 72%; (i) (iPr)_2_NP(Cl)OEtCN, CH_2_Cl_2_, 1.5 h, 71%; (j) solid phase DNA synthesis; (k) NH_3_, 16 h, 50°C; (l) TEA.3HF, 40°C, o.n.; (m) NaIO_4_, pH = 5.4.

The synthesis of the C3 precursor **8** started with a Sonogashira cross-coupling reaction between 7-Iodo-7-deazaguanosine (**5**) (17) with the protected alkynediol **4** (Figure 3). Note that **4** was synthesized starting from the protected (*S*)-glyceraldehyde **1**, which was converted to the dibromo-olefin **2** (38) and reprotected as TBDMS-ether **3**, compatible for solid phase DNA synthesis. Treatment of **3** with EtMgBr gave acetylene **4** (39). The alkyne group in **6** was reduced by passing hydrogen over palladium catalyst. Subsequently, protecting groups on the 5′- and 3′- positions of the sugar were exchanged using standard reagents and conditions to generate the phosphoramidite **8**. Using solid phase DNA synthesis, **8** was incorporated in two complementary 20-mer strands containing a 5′-G*NC sequence, where G* denotes the aldehyde-bearing residue. The oligonucleotides were deprotected—first with concentrated NH_3_ to remove the standard base and phosphate protecting groups and then with TEA·3HF to remove the TBDMS groups, generating the free diol. The oligonucleotides were purified by HPLC and the presence of the diol in the modified residues was confirmed by MALDI-TOF mass spectrometry measurements (see Supplementary Data). In addition, 20-mer oligonucleotides with the C1 and C2 aldehyde precursors on complementary strands were also synthesized and purified according to our earlier published protocol (17,22).

### ICL formation using three different length alkyl aldehyde precursors

**Figure 4.**
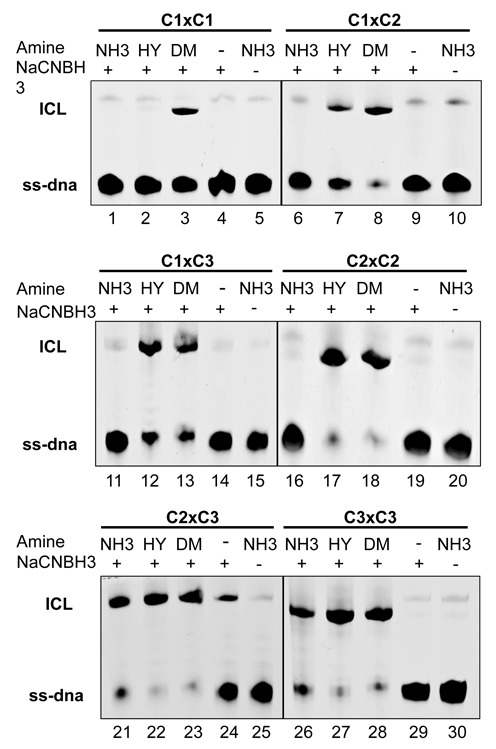
ICL formation by reductive amination using C1, C2 and C3 precursors. Analysis of ICL formation of the reaction of duplexes containing C1, C2 and C3 precursors in the presence of amines and NaBH_3_CN. Reactions were analyzed by denaturing polyacrylamide gel electrophoresis and stained with methylene blue. The sequences used were 5′- d(GTCACTGGTAG*ACAGCATTG) and 5′-d(CAATGCTG*TCTACCAGTGAC), where G* represents the modified guanine having seither C1, C2 or C3 diol. As a control for each set of ICL formation, reactions in which the amine or NaBH_3_CN was omitted were performed. The precursor and amine used are indicated over each gel. NH_3_, ammonia; HY, hydrazine; DM, DMEDA.

We studied ICL formation using the three different length precursors (C1, C2 and C3) and three amines (ammonia, hydrazine and DMEDA) in a systematic manner (Figure 4). ICLs were in 5′-G*NC sequences, the preferred site of ICL formation by nitrogen mustards (40,41). As previously discussed, the efficiency of ICL formation is dependent on both the reactivity of the amine as well as the distortion induced in the DNA. Duplex bearing diol-containing ICL precursors were annealed, the diol oxidized to the aldehyde using sodium periodate and ICL formation induced by incubation with one of the three amines in the presence of sodium cyanoborohydride. The identity of the ICLs formed was confirmed by MALDI-TOF measurements (see Supplementary Data). With a duplex having C1 precursors on each strand, ICL formation, indicated by the appearance of a band with slower mobility on a denaturing polyacrylamide gel, was only observed with DMEDA, yielding a 8.4 Å linkage (Figure 4, lanes 3). By contrast, we did not observe the formation of the 4.8 Å or 6.0 Å ICLs with two C1 precursors and ammonia and/or hydrazine, respectively (Figure 4, lanes 1 and 2). ICL formation was dependent on the presence of the reducing agent and the amine and omission of either one prevented ICL formation (Figure 4, lanes 4 and 5). In a duplex containing a C1 and a C2 aldehyde precursor on complementary strands, ICL formation was observed with hydrazine and DMEDA (Figure 4, lanes 7 and 8), but not with ammonia (Figure 4, lane 6), as previously reported (22). The 7.2 Å ICL containing hydrazine is the shortest ICL that we were able to form in our approach and it is isosteric to the native nitrogen mustard ICL. Having two C2 precursors gave ICLs with hydrazine and DMEDA (Figure 4, lanes 17 and 18), but not with ammonia (Figure 4, lane 16). These results confirmed that an ICL isostructural to that formed by NM with a linker length of 7.2 Å and inducing a bend in the DNA of about 20° can be formed by double reductive amination with hydrazine, but not ammonia, in line with the higher reactivity of hydrazine.

The reactivity patterns of ICL formation were further investigated with the newly synthesized C3 aldehyde ICL precursor paired to a complementary strand with C1 or C2 precursor. As was expected from the results with the C2/C2 precursors (Figure 4, lanes 16–18), ICL formation with the C1/C3 pair did not yield an ICL upon reaction with ammonia, showing that a 7.2 Å ICL cannot be formed with ammonia, irrespective of the position of carbon and nitrogen atoms in the ICL. As expected, ICLs were formed with C1/C3 using hydrazine and DMEDA (Figure 4, lanes 12 and 13) as an amine.

The question then arose whether the combination of C3 and C2 precursors would be able to yield an ICL with NH_3_, with an 8.4 Å linkage. This was indeed the case, and ammonia, hydrazine and DMEDA all formed ICLs with the C2/C3 oligonucleotides (Figure 4, lanes 21–23). This result shows that the minor distortion in the DNA of the 8.4 Å ICL allowed the reductive amination to occur with ammonia. A band of lesser intensity comigrating with the ICL band was also formed in the absence of an amine with the C2/C3 duplex (Figure 4, lane 24). Although the identity of this band remains to be determined, we speculate that it is the result of a reductive amination reaction of one of the aldehyde group with an exocyclic amine in the complementary strand of DNA. As expected based on these observations, reactions of the duplex containing two C3 aldehyde precursors, yielded ICL with all of the three amines with lengths of 9.6 Å and more (lanes 26–28, Figure 4).

### Summary and conclusion: Distance-dependent ICL formation

**Figure 5.**
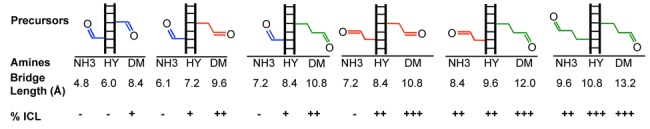
Overview of the reaction with of all the ICL reactions tested. Reactions with the C1, C2 and C3 precursors and the three different amines (NH_3_, HY and DMEDA), the theoretical bridge lengths and relative qualitative yields are indicated. ‘+’ low yield, ‘++’ moderate yield and ‘+++’ for high yield. As staining with methylene blue does not allow for the quantitative determination of the ratio of ss- versus ds-DNA, qualitative yields are indicated.

**Figure 6.**
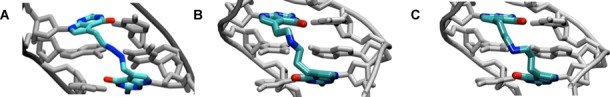
Models of structures of nitrogen mustard ICLs. **A.** C1/C2/HY ICL isosteric to a native nitrogen mustard ICL with a bridge length of 7.2 Å inducing a 20° bend in the DNA as predicted by molecular modeling studies (22); **B.** C2/C3/NH_3_ ICL with a bridge length of 8.4 Å, inducing a distortion in the DNA. **C**. C3/C3/NH_3_ ICL with a bridge length of 9.6 Å without DNA distortion. The structure in (**A**) was calculated using molecular dynamic simulations (22), (**B**) and (**C**) were manually generated using VMD 1.9 (42).

We used oligonucleotides with 7-deazaguanine residues having alkyl aldehyde chains of different lengths (C1, C2, C3) at the 7 position and studied ICL formation with ammonia, hydrazine and DMEDA using a reductive amination reaction. The efficiency of ICL formation was found to be correlated with the length of the ICL and the reactivity of the amine (summarized in Figure 5). We were able to form ICLs with bridge lengths ranging from 7.2 Å, for which our molecular modeling studies predict a bend of about 20° in the DNA duplex (22), to those of 10.8 Å and more, which our preliminary nuclear magnetic resonance (NMR) experiments show are free of distortion (AG, T. Zaliznyak, C. de los Santos, ODS, unpublished data). ICL formation was found to be most efficient with nondistorting ICLs (10.8–13.2 Å), followed by those with minor (8.4–9.6 Å) and moderate distortion (7.2–8.4 Å) (Figure 5). The higher nucleophilicity and reactivity of hydrazine allowed for the formation of more distorted ICLs. The major groove ICLs reported here inducing no, minor and moderate distortion in DNA duplexes (Figure 6) will be invaluable for advancing studies elucidating structure–function relationships in ICL repair.

## SUPPLEMENTARY DATA

Supplementary Data are available at NAR Online.

SUPPLEMENTARY DATA
