# Supplementary material for: Synthesis of structurally diverse major groove DNA interstrand crosslinks using three different aldehyde precursors
Source: Nucleic Acids Res. 2014 Apr 29;42(11):7429–35. doi: 10.1093/nar/gku328 (PMC4066762; doi:10.1093/nar/gku328)
Supplement: SUPPLEMENTARY DATA [file supp_42_11_7429__index.html]

Synthesis of structurally diverse major groove DNA interstrand crosslinks using three different aldehyde precursors — Synthesis of structurally diverse major groove DNA interstrand crosslinks using three different aldehyde precursors — SUPPLEMENTARY DATA 

# Synthesis of structurally diverse major groove DNA interstrand crosslinks using three different aldehyde precursors

## SUPPLEMENTARY DATA

**Files in this Data Supplement:**

- SUPPLEMENTARY DATA
