# Supplementary material for: Synthesis of structurally diverse major groove DNA interstrand crosslinks using three different aldehyde precursors
Source: Nucleic Acids Res. 2014 Apr 29;42(11):7429–35. doi: 10.1093/nar/gku328 (PMC4066762; doi:10.1093/nar/gku328)

Supplementary Information

for:

Synthesis of Structurally Diverse Major Groove DNA Interstrand Crosslinks Using Three Different Aldehyde Precursors

Shivam Mukherjee[1], Angelo Guainazzi[2,3], and Orlando D. Schärer[1,2*]

1. Department of Chemistry, Stony Brook University, Stony Brook, NY 11794-3400, USA

2. Department of Pharmocological Sciences, Stony Brook University, Stony Brook, NY 11794-8651, USA.

3. Present address: Helsinn Therapeutics (U.S.) Inc., Bridgewater, NJ 08807, USA.

*To whom correspondence should be addressed: Tel: +1-631-632-7545; Fax: +1-631-632-7546; Email: orlando.scharer@stonybrook.edu.

Table of Contents

General Information	1
Experimental Procedure for synthesized compounds	2
Supplementary References	10
NMR Spectra of the synthesized compounds	11
Table showing the calculated m/z values for the synthesized ICLs	19
MALDI-TOF Spectra of the synthesized ICLs	20

General Information

6-chloro-7-deaza-7-iodo-N(2)-isobutyryl-3’,5’-di-O-p-toluoyl-2’-deoxyguanosine was prepared according to published procedures (1). 4,4’-Dimethoxytrityl chloride was purchased from Syngen (USA). 2-cyanoethyl-N,N-diisopropylchlorophosphoramidite was purchased from ChemGenes (USA). Protected 2’-deoxyribonucleoside-3’-phosphoramidites and all other reagents necessary for automated DNA synthesis were purchased from Glen Research (USA). All other reagents and solvents were purchased from Sigma Aldrich (USA), EMD (USA) or Alpha Aesar. Dry solvents (acetonitrile, dichloromethane, dioxane, DMF, ethyl acetate, methanol, pyridine and THF) were purchased from EMD and used without further purification. NMR Spectra were recorded on Varian 400 MHz spectrometer. HR-MS were recorded on Thermo LTQ Orbitrap XL Ion Trap Mass Spectrometer. MALDI-TOF were recorded on Voyager-DE STR. All reactions were carried out under an inert atmosphere of nitrogen or argon. Chemical shifts (δ) are in ppm. J values are in Hz. **1** and **2** were synthesized according to published literature procedures (2).

(*R*)-3,4-di-(O-tertbutyl-dimethylsilyl)but-(1,1-dibromo)-ene (3)

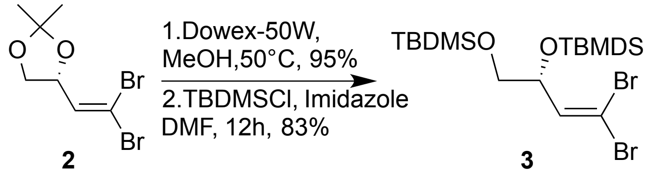

To a solution of (*R*)-4-(2,2-Dibromoethenyl)-2,2-dimethyl-1,3-dioxolane (4 g, 13.9 mmol) in 40 mL methanol was added Dowex-50W (4.4 g) and the resulting mixture was refluxed at 50 °C for 12 h. Dowex-50W was filtered off and methanol was evaporated. The resulting liquid was dissolved in DMF (45 mL) and treated with TBDMSCl (50% w/w in toluene, 14 ml, 40 mmol) and imidazole (5.4 g, 80 mmol). The reaction was incubated at room temperature for 12 h, diluted with 400 mL ethyl acetate, washed with 5% HCl (400mL), sat. $NaHCO_3$ (400 mL), and brine (300 mL), dried over Na_2SO_4, filtered and evaporated to dryness under reduced pressure. Purification of the residue by silica gel column chromatography (hexane/ethyl acetate 4:1) gave the product as a colorless liquid (6 g, 12.6 mmol, 90%).

R_f : 0.30 (hexane/ethyl acetate 4:1)

^{1}H NMR ($CDCl_3$): δ 6.33 (d, 1H, C(2)-H, J = 8.4), 4.34 (m, 1H, C(4)-H_a), 3.56 (dd, 1H, C(3)-H, J = 10.4, 6), 3.48 (dd, 1H, C(4)-H_b, J = 10.4, 6), 0.89 & 0.87 (2s, 18H, tBu-Me), 0.08 & 0.07 (2s, 6H, Si-Me), 0.05 (s, 6H, Si-Me).

^{13}C NMR: ($CDCl_3$): δ 140.0 (C), 89.9 (C), 74.4 (CH_2), 66.2 (CH), 25.9 (CH_3), 25.7 (CH_3), 18.3 (C), 18.1 (C), - 4.5 (CH_3), - 5.3 (CH_3).

ESI-MS: $[M+H]^+$ m/z calculated for $C_{10}H_{20}Br_2OSi$ 341.96, observed 341.0 (fragmented during MS).

(*R*)-3,4-di-(O-tertbutyl-dimethylsilyl)but-1-yne (4)

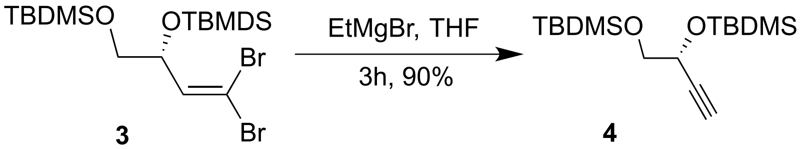

3 4

To a solution of (*R*)-3,4-di-(O-tertbutyl-dimethylsilyl)but-(1,1-dibromo)-ene (3 g, 6.32 mmol) in THF (10 mL) cooled in an ice bath was added EtMgBr (1M in THF, 12.6 mL, 12.6 mmol) dropwise. The ice bath was removed and the reaction mixture was stirred at room temperature for 3 h. Solid NH_4Cl (0.8 g) was added to quench excess of EtMgBr, and the reaction mixture was diluted with ethyl acetate (40 mL), washed with brine (40 mL), dried over Na_2SO_4, filtered and evaporated to dryness under reduced pressure. Purification of the residue by silica gel column chromatography (hexane/ethyl acetate 4:1) gave the product as colorless liquid (1.4 g, 4.4 mmol, 70%).

R_f : 0.65 (hexane/ethyl acetate 4:1)

^{1}H NMR ($CDCl_3$): δ 4.37 (m, 1H, C(4)-H_a), 3.69-3.66 (m, 2H, C(4)-H_b & C(3)-H), 2.36 (d, 1H, C(1)-H, J = 2.4), 0.91 & 0.90 (2s, 18H, tBu-Me), 0.14 & 0.12 (2s, 6H, Si-Me), 0.08 & 0.07 (2s, 6H, Si-Me).

^{13}C NMR: ($CDCl_3$): δ 83.7 (C), 72.9 (CH), 68.1 (CH_2), 64.7 (CH), 26.1 (CH_3), 25.9 (CH_3), 18.5 (C), 18.4 (C), - 4.6 (CH_3), -5.1 (CH_3).

HR ESI-MS: $[M+H]^+$ m/z calculated for $C_{16}H_{35}O_2Si_2$ 315.2170, observed 315.2169.

7-deaza-7-iodo-N(2)-isobutyryl-3’,5’-di-O-p-toluoyl-2’-deoxyguanosine (5)

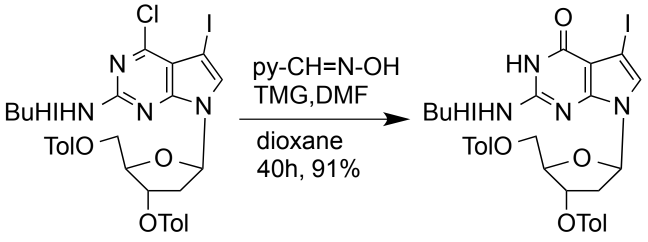

5

A solution of 6-chloro-7-deaza-7-iodo-N(2)-isobutyryl-3’,5’-di-O-p-toluoyl-2’-deoxyguanosine (3.7 g, 5.16 mmol), pyridine-2-carboaldoxime (3.15 g, 25.8 mmol) and 1,1,3,3-tetramethylguanidine (3.26 mL, 25.8 mmol) in DMF (60 mL) and dioxane (60 mL) was stirred for 40 h at room temperature. The reaction mixture was diluted with ethyl acetate (200 mL) and washed with 5% HCl (200 mL), sat. $NaHCO_3$ (200 mL) and brine (100 mL), dried over Na_2SO_4, filtered and evaporated to dryness under reduced pressure. Purification of the residue by silica gel column chromatography (hexane/ethyl acetate 2:1) gave the product as a light yellow solid (3.3 g, 4.72 mmol, 91 %).

R_f : 0.75 (hexane/ethyl acetate 1:1)

^{1}H NMR ($CDCl_3$): δ 11.64 (s, 1H, N(1)-H), 8.64 (s, 1H, C2-(NH)), 7.93-7.87 (dd, 4H, Tol-H, J = 6.8, 2), 7.27-7.23 (dd, 4H, Tol-H, J = 10, 7.6), 6.92 (s, 1H, C(8)-H), 6.23 (dd, 1H, C(1’)-H, J = 14, 7.6), 5.79 (dd, 1H, C(3’)-H, J = 6, 2.8), 4.99 (dd, 1H, C(5’)-H_a, J = 10.8, 6.8), 4.62- 4.57 (m, 2H, C(5’)-H_b & C(4’)-H), 3.05-2.98 (m, 1H, C(2’)-H_a), 2.67 (sept, 1H, iBu-CH, J = 6.8), 2.59 (ddd, 1H, C(2’)-H_b, J = 14.4, 6.4, 2.8), 2.43 & 2.41 (2s, 6H, Tol-Me), 1.31-1.28 (d, 6H, iBu-Me, J = 7.2).

^{13}C NMR: ($CDCl_3$): δ 178.2 (C), 166.5 (C), 165.7 (C), 156.9 (C), 147.0 (C), 146.4 ©, 144.2 (C), 129.5 (CH), 129.5 (CH), 129.3 (CH), 129.2 (CH), 129.1 (CH), 126.4 (CH), 126.2 (CH), 105.9 (C), 85.0 (CH), 81.9 (CH), 74.7 (CH_2), 63.4 (CH_2), 55.1 (CH), 37.2 (CH_2), 36.1 (CH_2), 21.5 (CH_3), 18.8 (C).

HR ESI-MS: $[M+H]^+$ m/z calculated for $C_{31}H_{32}O_7N_4I$ 699.1310, observed 699.1306.

7-deaza-7-(*S*)-(3,4-di-(O-tertbutyl-dimethylsilyl)butynl)-N(2)-isobutryl-3'-5'-di-O-p-toluoyl-2'-deoxyguanosine (6)

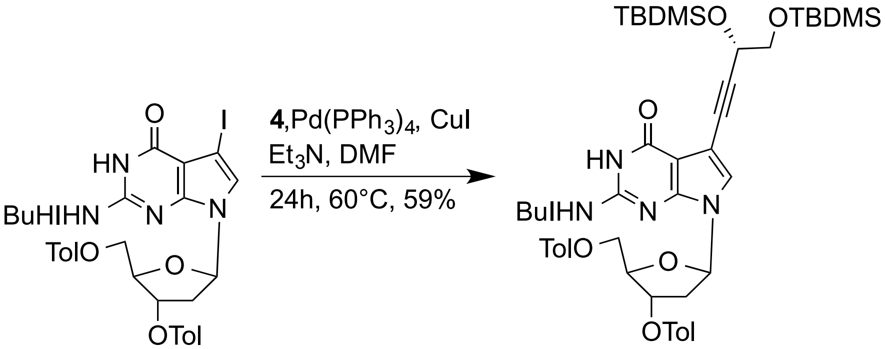

5 6

To a solution of 7-deaza-7-iodo-N(2)-isobutyryl-3',5'-di-O-p-toluoyl-2'-deoxyguanosine (1 g, 1.43 mmol), Pd[$(PPh)_3$]$_4$ (160 mg, 0.14 mmol), CuI (50 mg, 0.28 mmol), Et_3N (0.4 mL, 2.86 mmol) in DMF (16 mL) was added **4** (1.35 g, 4.29 mmol) and the reaction mixture was stirred at 60 $^{\circ}$C for 24 h, diluted with ethyl acetate (150 mL) and washed with 5% HCl (150 mL), sat.$NaHCO_3$ (150 mL), brine (100 mL), dried over Na_2SO_4, filtered and evaporated to dryness under reduced pressure. Purification of the residue by silica gel column chromatography (hexane/ethyl acetate 3:1) gave the product as a yellow solid (750 mg, 0.84 mmol, 59%).

R_f: 0.80 (hexane/ethyl acetate 1:1)

^{1}H NMR ($CDCl_3$): δ 11.62 (s, 1H, N(1)-H), 8.70 (s, 1H, C2-(NH)), 7.91 (d, 2H, Tol-H, J = 8.8), 7.86 (d, 2H, Tol-H, J = 8.4), 7.27-7.21 (dd, 4H, Tol-H, J = 16.8, 8.4), 6.99 (s, 1H, C8-(H)), 6.19 (t, 1H, C(1')-H, J = 6.8), 5.81 (dd, 1H, C(3')-H, J = 6, 2.4), 5.06 (dd, 1H, C(5')-H, J = 10.8, 5.6), 4.62 (m, 3H, C(5')H_b, C(4')H, C(7)-C≡C-C(*H*)-O-), 3.78 (dd, 1H, C(7)-C≡C-$CH_{2(a)}$, J = 10.4, 4), 3.70 (dd, 1H, C(7)-C≡C-$CH_{2(b)}$, J = 10.4, 8), 3.10 (dd, 1H, C(2')H_a, J = 14.4, 6.4), 2.71 (sept, 1H, iBu-CH, J = 6.8), 2.56 (ddd, 1H, C(2')H_b, J = 14.4, 6.4, 2.8), 2.43 (s, 3H, Tol-Me), 2.40 (s, 3H, Tol-Me), 1.30 (dd, 6H, iBu-Me, J = 8, 6.8), 0.92 & 0.89 (2s, 18H, t-BuMe), 0.18 & 0.17 (2s, 6H, Si-Me), 0.07 (2s, 6H, Si-Me).

^{13}C NMR: ($CDCl_3$): δ 178.5 (C), 165.8 (C), 156.5 (C), 146.7 (C), 146.3 (C), 144.3 (C), 129.6 (C), 129.2 (C), 129.1 (C), 105.8 (C), 100.6 (C), 90.1 (C), 86.0 (CH), 81.8 (CH), 74.8 (CH), 68.1 & 65.48 (-C≡C-), 63.5 (CH_2), 36.8 (CH_2), 36.3 (CH), 25.9 (CH_3), 25.8 (CH_3), 18.9 (C), 18.3 (C), - 4.6 (CH_3), - 4.8 (CH_3), -5.2 (CH_3), -5.3 (CH_3).

HR ESI-MS: $[M+H]^+$ m/z calculated for $C_{47}H_{65}O_9N_4Si_2$ 885.4284, observed 885.4290.

7-deaza-7-(*S*)-(3,4-di-(O-tertbutyl-dimethylsilyl)butyl)-N(2)-isobutryl-3'-5'-di-O-p-toluoyl-2'-deoxyguanosine

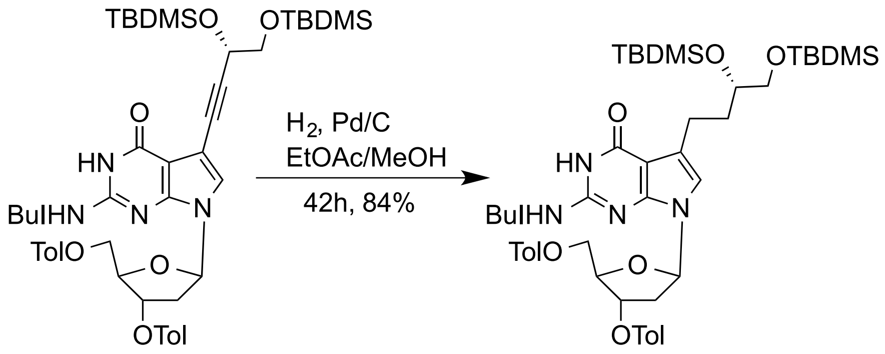

6

To a solution of 7-deaza-7 -(*S*)-(3,4-di-(O-tertbutyl-dimethylsilyl)butynl)-N(2)-isobutryl-3'-5'-di-O-p-toluoyl-2'-deoxyguanosine (450 mg, 0.5 mmol) in 10 mL EtOAc and 10 mL MeOH was added 10% Pd/C (100 mg). The reaction was monitored using mass spectrometry for complete reduction of the triple bond. After the reduction was complete (42 h), the solution was filtered over Celite and solvent evaporated under reduced pressure. Purification of the residue by silica gel column chromatography (hexane/ethyl acetate 2:1) gave the product as a light yellow solid (380 mg, 0.42 mmol, 84 %).

R_f : 0.57 (hexane/ethyl acetate 2:1)

^{1}H NMR ($CDCl_3$): δ 11.53 (s, 1H, N(1)-H), 8.58 (s, 1H, C2-(NH)), 7.93 (d, 2H, TolH, J = 8), 7.88 (d, 2H, TolH, J = 8), 7.27-7.21 (dd, 4H, J = 12.8 ,8), 6.56 (s, 1H, C8-(H)), 6.24 (dd, 1H, C(1')-H, J = 7.6, 6.4), 5.80 (t, 1H, C(4')-H, J = 2.8), 5.04 (dd, 1H, C(5')-H_a, J = 10.0, 4.4), 4.60 (m, 2H, C(5')-H_b & C(3')-H), 3.73 (m, 1H, C(7)-$(CH)_2$-C(*H*)-O-), 3.55-3.47 (m, 2H, C(7)-$(CH)_2$-C$H_{2(a\&b)}$), 3.09 (dd, 1H, C(2')-H_a, J = 14.4, 6.4), 2.88-2.66 (m, 3H, C(7)-$CH_{2a\&b}$, iBu-CH), 2.51 (ddd, 1H, C(2')-H_b J = 14.4, 6.4, 2.8), 2.43&2.40 (2 s, 6H, Tol-Me), 1.95-1.86 (m, 1H, C(7)-$(CH_2)_2$-CH_a), 1.73-1.64 (m, 1H, C(7)-$(CH_2)_2$-CH_b), 1.28 (dd, 6H, iBu-Me, J = 16, 9.2), 0.88 & 0.87 (s, 9H, tBu-Me), 0.07 & 0.06 (2s, 6H, Si-Me), 0.03 (s, 6H, Si-Me).

^{13}C NMR: ($CDCl_3$): δ 178.3 (C), 166.7 (C), 165.8 (C), 157.7 (C), 146.9 (C), 145.7 (C), 144.2 (C), 129.6 (C), 129.1 (C), 126.5 (C), 121.7 (C), 116.5 (C), 105.2 (C), 85.5 (CH), 81.5 (CH), 75.0 (CH), 73.1 (CH), 63.7 (CH_2), 67.5 (CH_2), 36.6 (CH_2), 36.3 (CH), 34.4 (CH_2), 25.9 (CH_3), 21.6 (CH_3), 18.9 (C), 18.3 (C), - 4.2 (CH_3), - 4.6 (CH_3), - 5.3 (CH_3).

HR ESI-MS: $[M+H]^+$ m/z calculated for $C_{47}H_{69}O_9N_4Si_2$ 889.4597, observed 889.4602.

7-deaza-7-(*S*)-(2,3-di-(O-tertbutyl-dimethylsilyl)butyl)-N(2)-isobutryl-2'-deoxyguanosine (7)

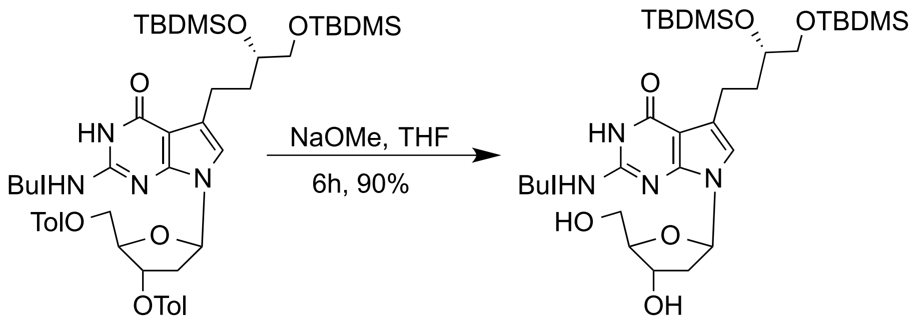

7

To a solution of 7-deaza-7-(*S*)-(3,4-di-(O-tertbutyl-dimethylsilyl)butyl)-N(2)-isobutryl-3'-5'-di-O-p-toluoyl-2'-deoxyguanosine (300 mg, 0.34 mmol) in 10 mL THF was added 1M NaOMe solution in MeOH (0.34 mL) in an ice bath. The ice bath was removed and the reaction mixture was allowed to run at room temperature for 6 h, quenched with 20 μL acetic acid and the solvent evaporated under reduced pressure. Purification of the residue by silica gel column chromatography (dichloromethane/methanol 20:1) gave the product as a white solid (200 mg, 0.30 mmol, 90%).

R_f : 0.71 (dichloromethane/methanol 10:1)

^{1}H NMR ($CDCl_3$): δ 11.59 (s, 1H, N1-(H)), 8.17 (s, 1H, C2-(NH)), 6.53 (s, 1H, C8-(H)), 6.09 (dd, 1H, C(1')-H, J = 9.2, 5.6), 4.65 (d, 1H, C(4')-H, J = 5.6), 4.12 (d, 1H, C(7)-(CH_2)-C(H)-$CH_{2(a)}$, J = 2), 3.90 (dd, 1H, C(7)-(CH_2)$_2$-CH-$CH_{2(b)}$, J = 12, 2.4), 3.73 (m, 2H, C(5')-H_a & C(3')-H), 3.54 (dd, 1H, C(5')-H_b, J = 10.4, 6), 3.47 (dd, 1H, C(7)-(CH_2)$_2$-C(*H*)-O-, J = 10, 5.6), 2.72 (m, 3H, iBu-CH & C(7)-C$H_{2(a\&b)}$), 2.56 (dd, 1H, C(2')-H_a, J = 13.6, 6.8), 2.23 (ddd, 1H, C(2')-H_b, J = 13.6, 5.6, 1.6), 1.89 (m, 1H, C(7)-CH_2-CH_{2a}), 1.66 (m, 1H, C(7)-CH_2-CH_{2b}), 1.24 (dd, 6H, iBu-Me, J = 6.8, 5.2), 0.89 & 0.88 (2 s, 18 H, tBu-Me), 0.07 & 0.06 (2s, 6H, Si-Me), 0.04 & 0.03 (2s, 6H, Si-Me).

^{13}C NMR: ($CDCl_3$): δ 179.2 (C), 158.1 (C), 147.1 (C), 146.1 (C), 121.4 (C), 116.7 (C), 104.9 (C), 87.1 (CH), 85.1 (CH), 72.9 (CH), 72.4 (CH), 67.5 (CH_2), 63.5 (CH_2), 39.9 (CH_2), 36.0 (CH_2), 34.5 (CH), 25.9 (CH_3), 21.9 (CH_3), 19.0 (C), 18.1 (C), - 4.2 (CH_3), - 4.6 (CH_3), - 5.3 (CH_3).

HR ESI-MS: $[M+H]^+$ m/z calculated for $C_{31}H_{57}O_7N_4Si_2$ 653.3760, observed 653.3760.

7-deaza-7-(*S*)-(2,3-di-(O-tertbutyl-dimethylsilyl)butyl)-5'-O-(4,4'-dimethoxytrityl)-N(2)-isobutryl-2'-deoxyguanosine

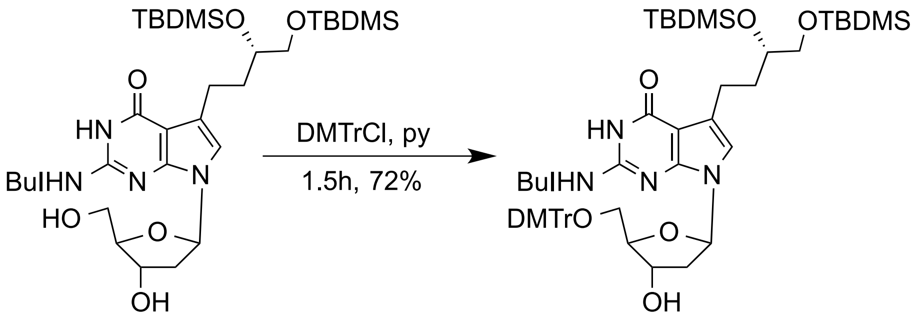

To a solution of 7-deaza-7-(*S*)-(3,4-di-(O-tertbutyl-dimethylsilyl)butyl)-N(2)-isobutryl-2'-deoxyguanosine (150 mg, 0.23 mmol) in 6 mL pyridine was added 4,4'-dimethoxytrityl chloride (90 mg, 0.26 mmol). The reaction was allowed to run at room temperature for 1.5 h, quenched with 60 µL of MeOH and pyridine evaporated under reduced pressure. Purification of the residue by silica gel column chromatography (hexane/ethyl acetate 1:1, 0.5% Et_3N) gave the product as a white solid (160 mg, 0.16 mmol, 72%).

R_f : 0.63 (hexane/ethyl acetate 1:2)

^{1}H NMR ($CDCl_3$): δ 11.52 (s, 1H, N(1)-H, 8.08 (s, 1H, C2-(NH)), 7.41 (dd, 2H, DMT-H, J = 6.4, 1.2), 7.31-7.20 (m, 7H, DMT-H), 6.80-6.77 (m, 4H, DMT-H), 6.55 (s, 1H, C8-(H)), 6.38 (dd, 1H, C(1')-H, J = 8, 5.6), 4.55 (m, 1H, C(4')-H), 4.04 (m, 1H, C7-$(CH_2)_2$-CH(O)-$CH_{2(a)}$), 3.76 (s, 6H, DMT-OMe), 3.70 (m, 1H, C7-(CH_2)-CH(O)-$CH_{2(b)}$), 3.52-3.43 (m, 2H, C(5')-H_a & C7-$(CH_2)_2$-C(*H*)-O), 2.79 (m, 1H, iBu-CH), 2.69 (m, 1H, C(2')-H_a), 2.57 (m, 1H, C7-$CH_{2(a)}$), 2.29-2.21 (m, 2H, C(2')-H_b & C7-$CH_{2(b)}$), 1.86-1.80 (m, 1H, C7-CH_2-CH_{2a}), 1.67-1.61 (m, 1H, C7-CH_2-CH_{2b}), 1.07 (dd, 6H, iBu-Me, J = 10.8, 6.8), 0.86 & 0.85 (2s, 18H, t-BuMe), 0.04 (s, 6H, Si-Me), 0.01 (s, 6H, Si-Me).

^{13}C NMR: ($CDCl_3$): δ 178.9 (C), 158.5 (C), 157.6 (C), 147.7 (C), 146.3 (C), 144.3 (C), 135.4 (C), 129.9 (C), 128.0 (C), 127.8 (C), 122.4 (CH), 114.4 (CH), 113.1 (CH), 104.1 (CH), 86.6 (C), 85.6 (CH), 82.6 (CH), 73.2 (CH), 67.6 (CH), 64.2 (CH), 55.0 (C), 39.6 (CH), 36.0 (CH_2), 34.7 (CH), 25.9 (CH_3), 22.2 (CH_2), 18.4 (CH_3), 18.3 (C), 18.1 (C), -4.2 (CH_3), -4.6 (CH_3), -5.3 (CH_3).

HR ESI-MS: $[M+H]^+$ m/z calculated for $C_{52}H_{75}O_9N_4Si_2$ 955.5067, observed 955.5069.

7-deaza-7-(*S*)-(3,4-di-(O-tertbutyl-dimethylsilyl)butyl)-5'-O-(4,4'-dimethoxytrityl)-N(2)-isobutryl-2'-deoxyguanosine-3'-[(2-cyanoethyl)N,N-diisopropylphosphoramidite] (8)

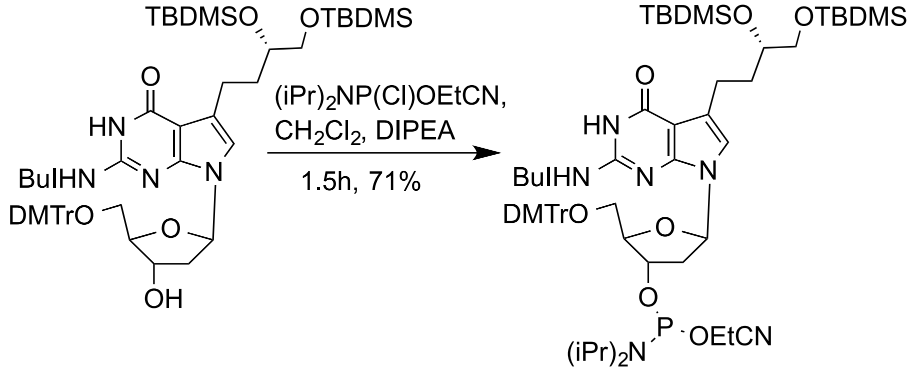

8

To a solution of 7-deaza-7-(*S*)-(2,3-di-(O-tertbutyl-dimethylsilyl)butyl)-5'-O-(4,4'-dimethoxytrityl)-N(2)-isobutryl-2'-deoxyguanosine () (160 mg, 0.17 mmol) in 4 mL CH_2Cl_2 and N-ethyl-diisopropylamine (117 μL, 0.67 mmol) was added 2-cyanoethyl-N,N-diisopropyl-chlorophosphoramidite (75 μL , 0.33 mmol). The reaction was stirred at room temperature for 1.5 h, quenched with 400 μL methanol and solvent evaporated under reduced pressure. Purification of the residue by silica gel column chromatography (hexane/ethyl acetate 3:1, 0.5% Et_3N) gave the product as a white solid (140 mg, 0.12 mmol, 71%).

R_f : 0.75 (hexane/ethyl acetate 1:2)

^{1}H NMR ($CDCl_3$): 11.51 (s, 1H, N(1)-H), 8.29 & 7.90 (2s, 1H, C2-(NH)), 7.47-7.43 (m, 2H, DMT-H), 7.35-7.19 (m, 7H, DMT-H), 6.80-6.76 (m, 4H, DMT-H), 6.59 (2 peaks, C8-(H)), 6.36-6.28 (m, 1H, C(1')-H), 4.71-4.60 (m, 1H, C7-$(CH_2)_2$-CH(O)-$CH_{2(a)}$), 3.88-3.80 (m, 1H, C7-$(CH_2)_2$-CH(O)-$CH_{2(b)}$), 3.77 & 3.76 (2s, 6H, DMT-OMe), 3.65-3.55 (m, 2H, iPr-CH), 3.54-3.44 (m, 2H, C(5')-H_a & C(3')-H), 3.37-3.22 (m, 2H, C(5')-H_b & C7-$(CH_2)_2$-C*H*), 2.86-2.77 (m, 1H, iBu-CH), 2.75-2.65 (m, 2H, C(2')-H_a & NC-CH_2), 2.63-2.51 (m, 1H, C7-$CH_{2(a)}$), 2.47 (t, 1H, NC-CH_2), 2.36-2.18 (m, 2H, C(2')H_b & C7-$CH_{2(b)}$), 1.88-1.82 (m, 1H, C7-$(CH_2)_2$-$CH_{2(a)}$), 1.69-1.58 (m, 1H, C7-$(CH_2)_2$-$CH_{2(b)}$), 1.19-1.05 (m, 18H, iPr-Me & iBu-Me), 0.86 (s, 18H, tBu-Me), 0.04 & 0.01 (2s, 12H, Si-Me).

^{31}P NMR ($CDCl_3$): 149.35, 149.22, 149.09, 148.93.

HR ESI-MS: $[M+H]^+$ m/z calculated for $C_{61}H_{92}O_{10}N_6PSi_2$ 1155.6145, observed 1155.6149.

References:

1. Angelov, T.; Guainazzi, A.; Schärer, O.D. (2009) Generation of DNA Interstrand Crosslinks by Post Synthetic Reductive Amination. *Org. Lett.*, **11**, 661-664.

2. Gooding, O.W.; Beard, C.C.; Jackson, D.Y.; Wren, D.L.; Cooper, G.F. (1991) Enantioselective Formation of Functionalized 1,3-Disubstituted Allenes: Synthesis of α- Allenic ω- Carbomethoxy Alcohols of High Optical Purity. *J. Org. Chem.*, 56, 1083-1088.

NMR Spectra

^{1}H & ^{13}C NMR Spectra of (*R*)-3,4-di-(O-tertbutyl-dimethylsilyl)but-(1,1-dibromo)-ene (3)

^{1}H

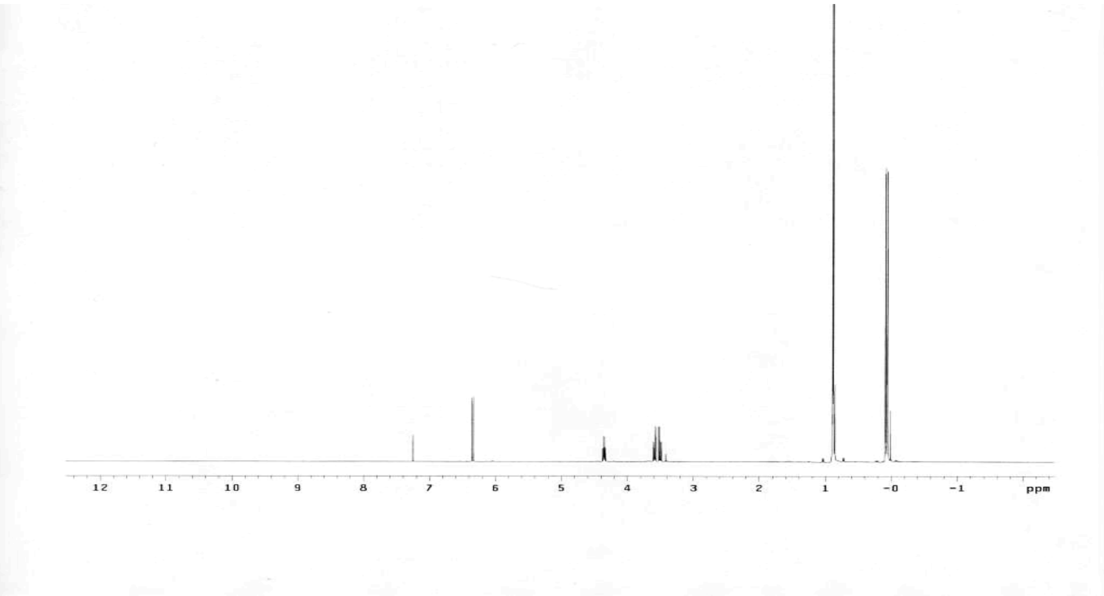

^{13}C

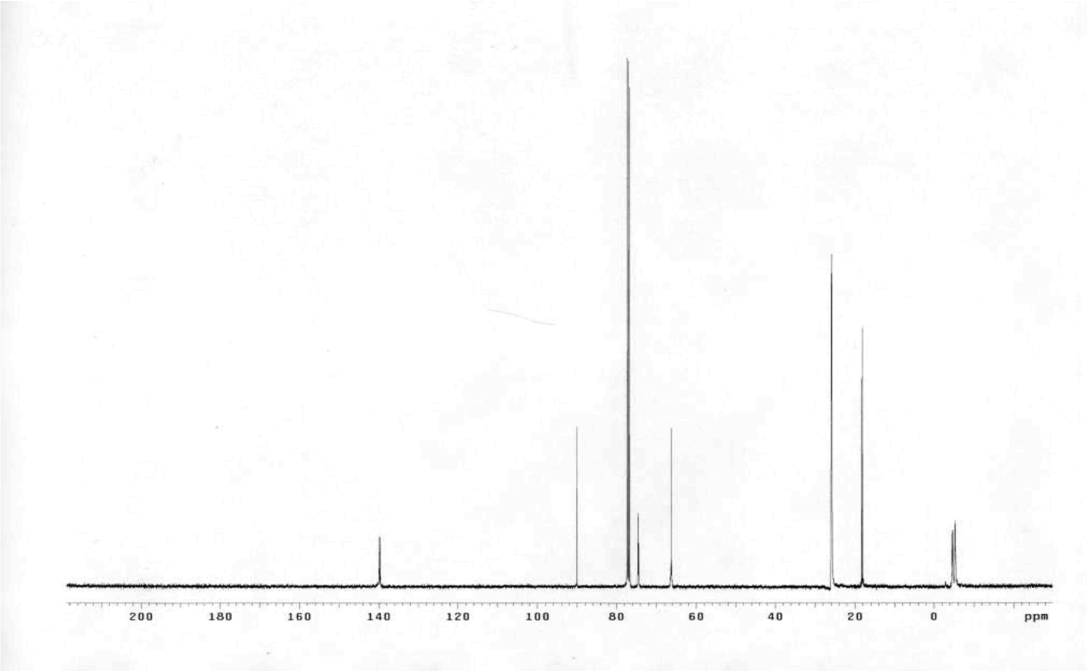

^{1}H & ^{13}C NMR Spectra of (*R*)-3,4-di-(O-tertbutyl-dimethylsilyl)but-1-yne (4)

[1]H

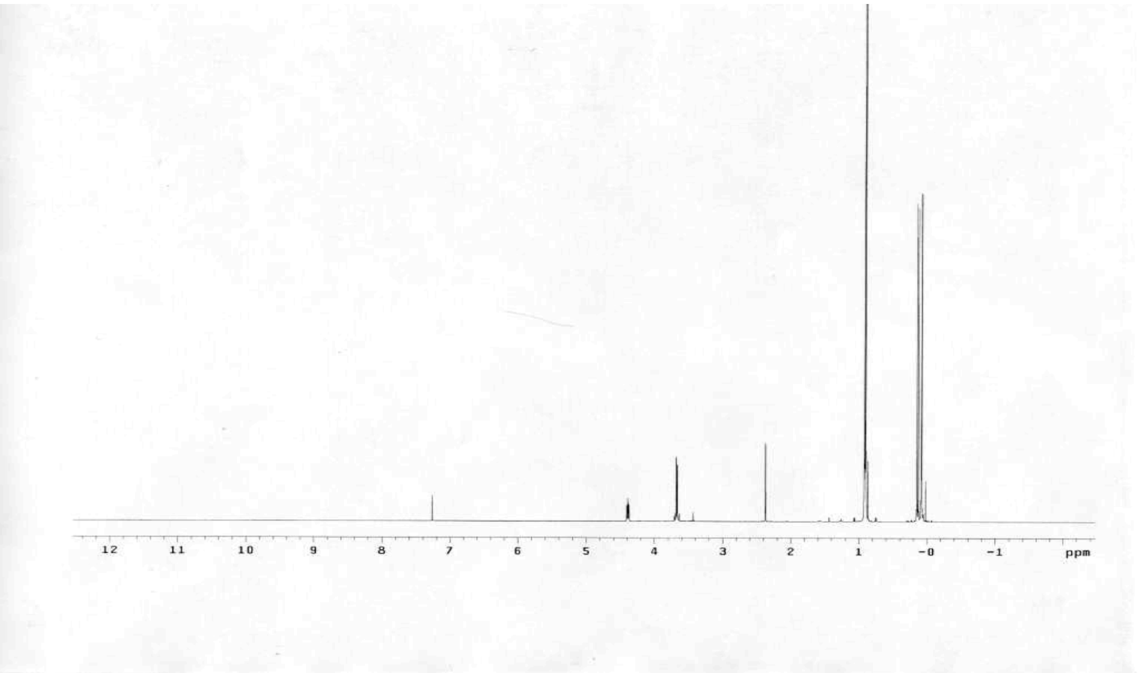

[13]C

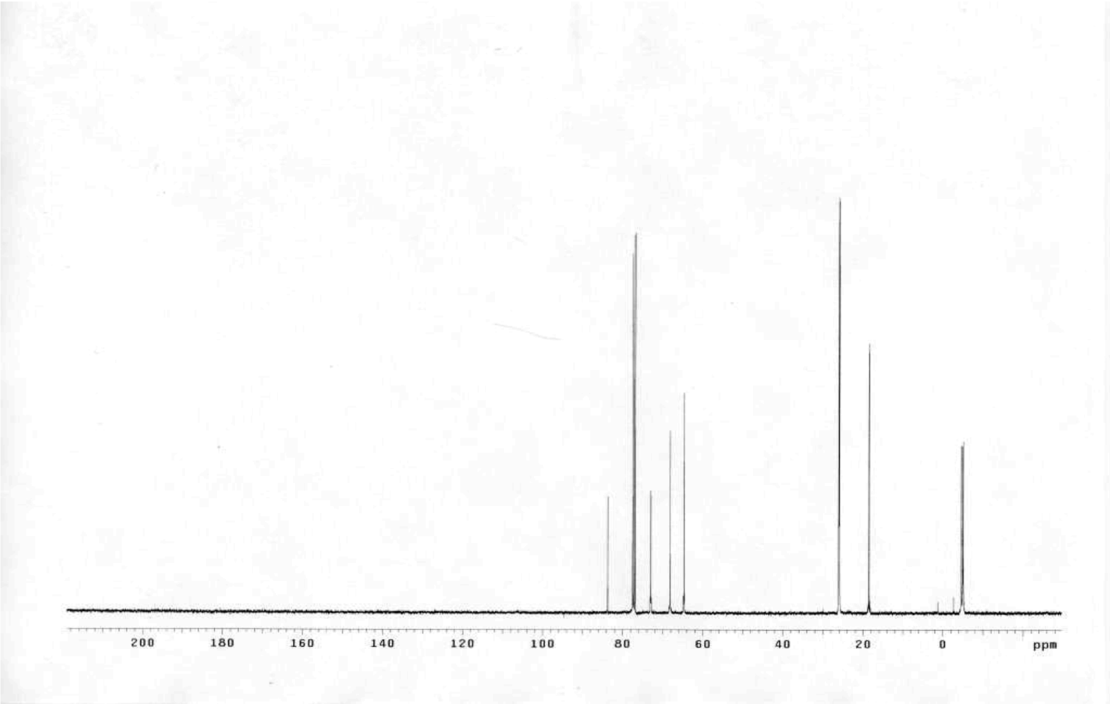

^{1}H & ^{13}C NMR Spectra of 7-deaza-7-iodo-N(2)-isobutyryl-3',5'-di-O-p-toluoyl-2'-deoxyguanosine (5)

^{1}H

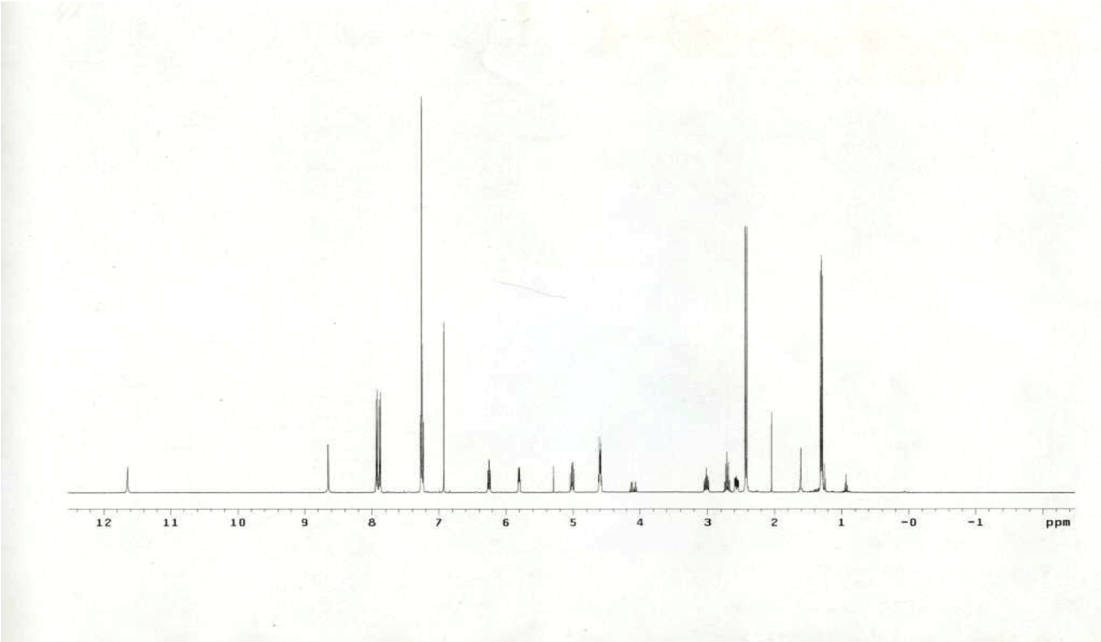

^{13}C

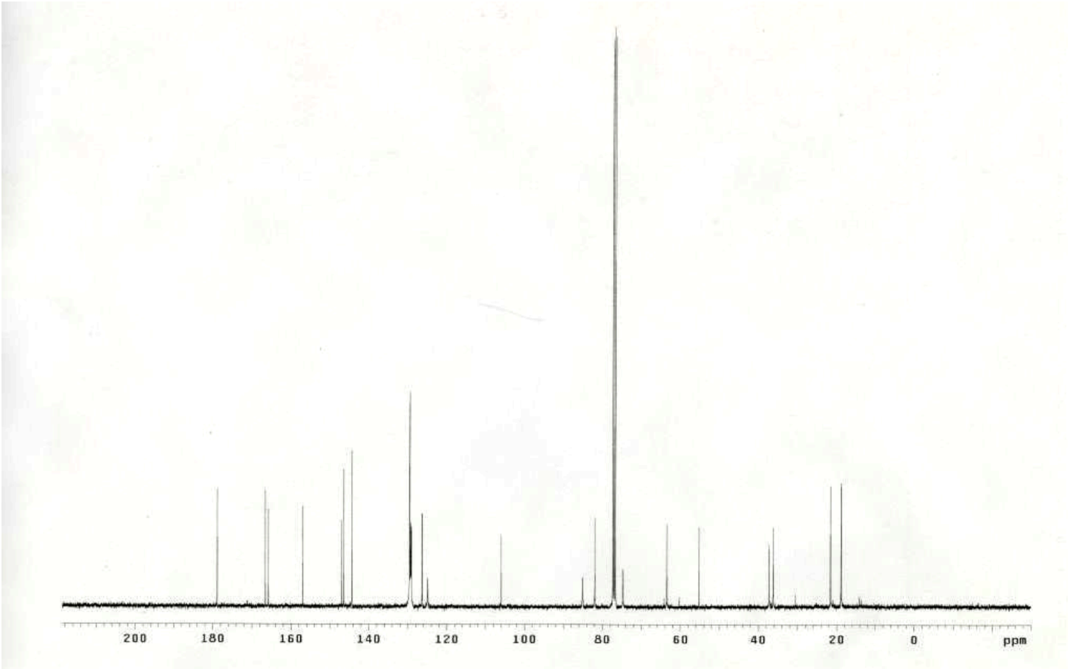

^{1}H & ^{13}C NMR Spectra of 7-deaza-7-(*S*)-(3,4-di-(O-tertbutyl-dimethylsilyl)butynl)-N(2)-isobutyryl-3',5'-di-O-p-toluoyl-2'-deoxyguanosine (6)

^{1}H

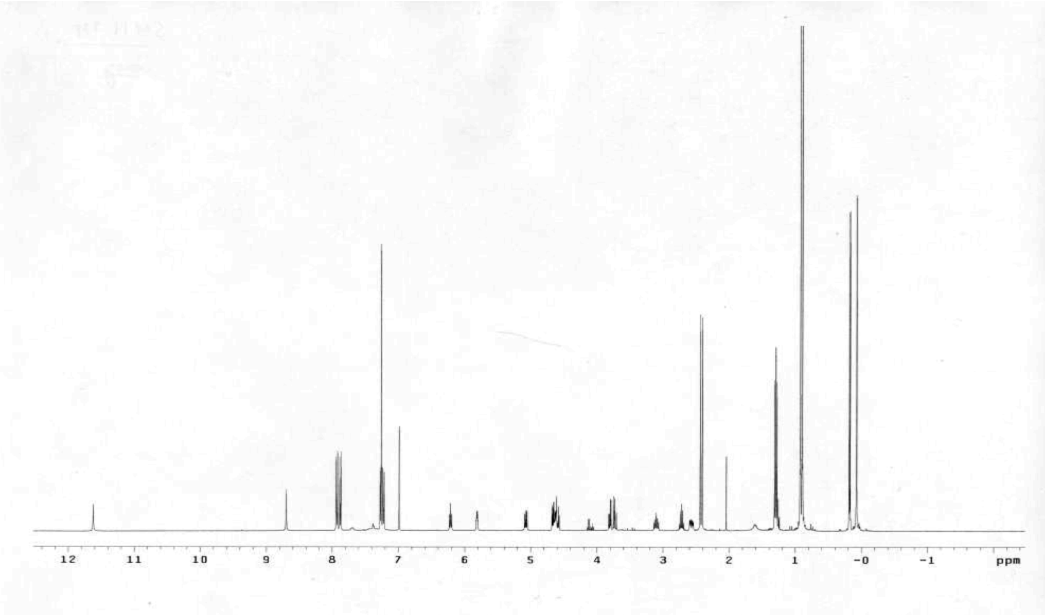

^{13}C

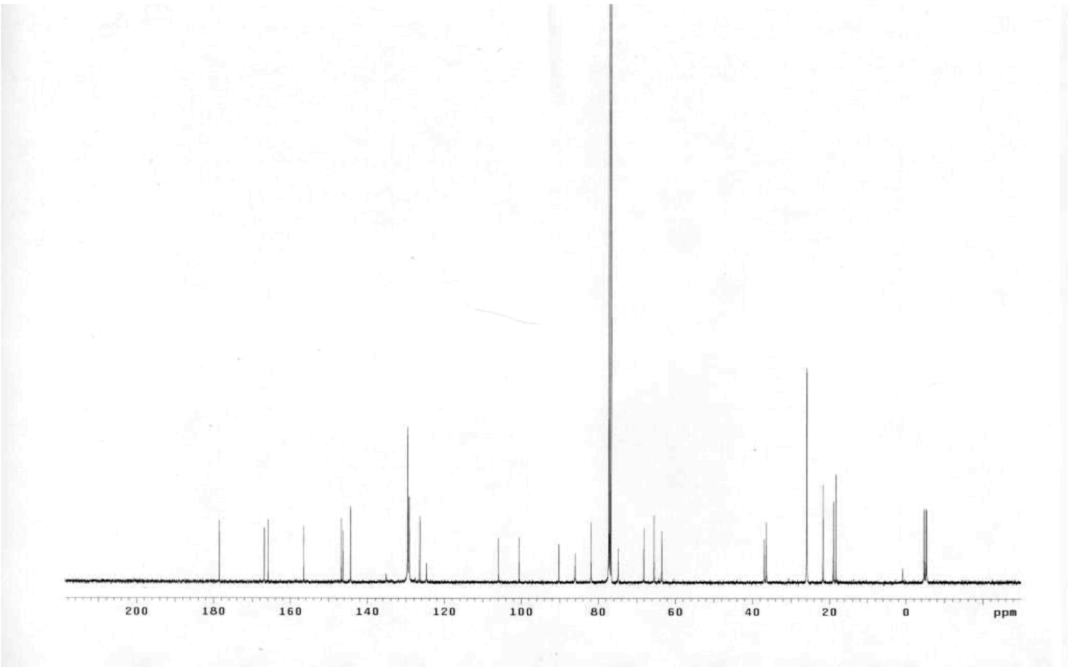

^{1}H & ^{13}C NMR Spectra of 7-deaza-7-(*S*)-(3,4-di-(O-tertbutyl-dimethylsilyl)butyl)-N(2)-isobutyryl-3',5'-di-O-p-toluoyl-2'-deoxyguanosine

[1]H

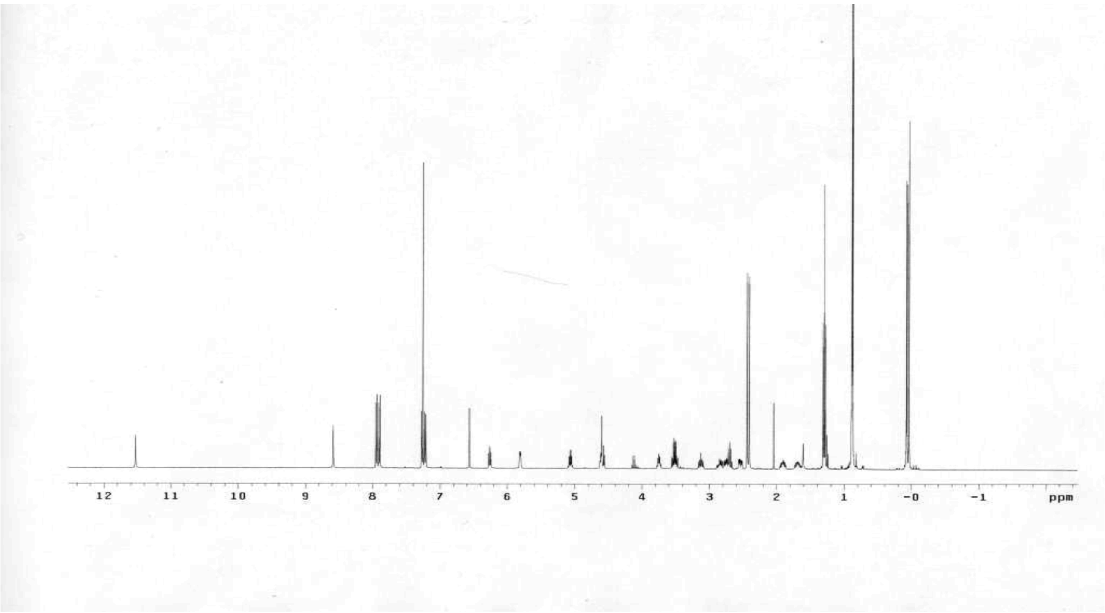

[13]C

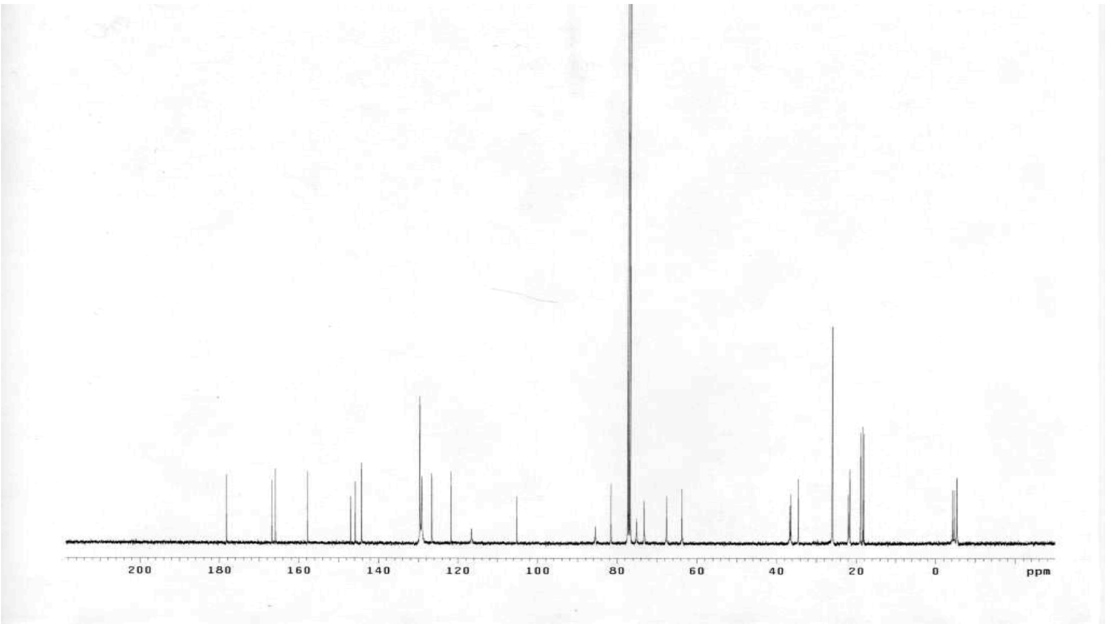

^{1}H & ^{13}C NMR Spectra of 7-deaza-7-(*S*)-(3,4-di-(O-tertbutyl-dimethylsilyl)butyl)-N(2)-isobutyryl-2'-deoxyguanosine (7)

^{1}H

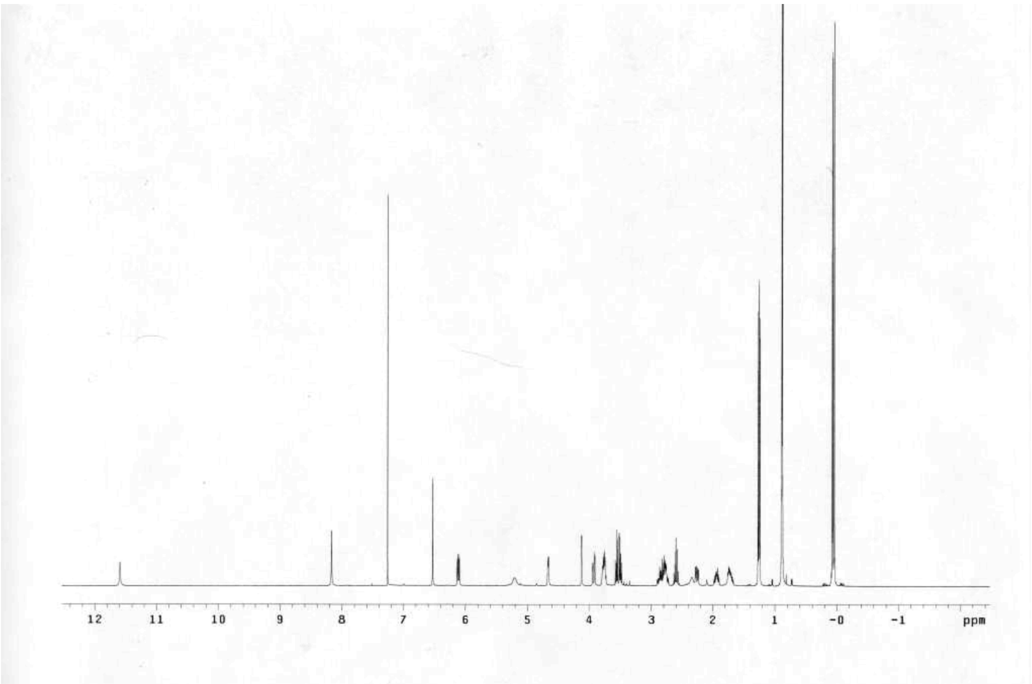

^{13}C

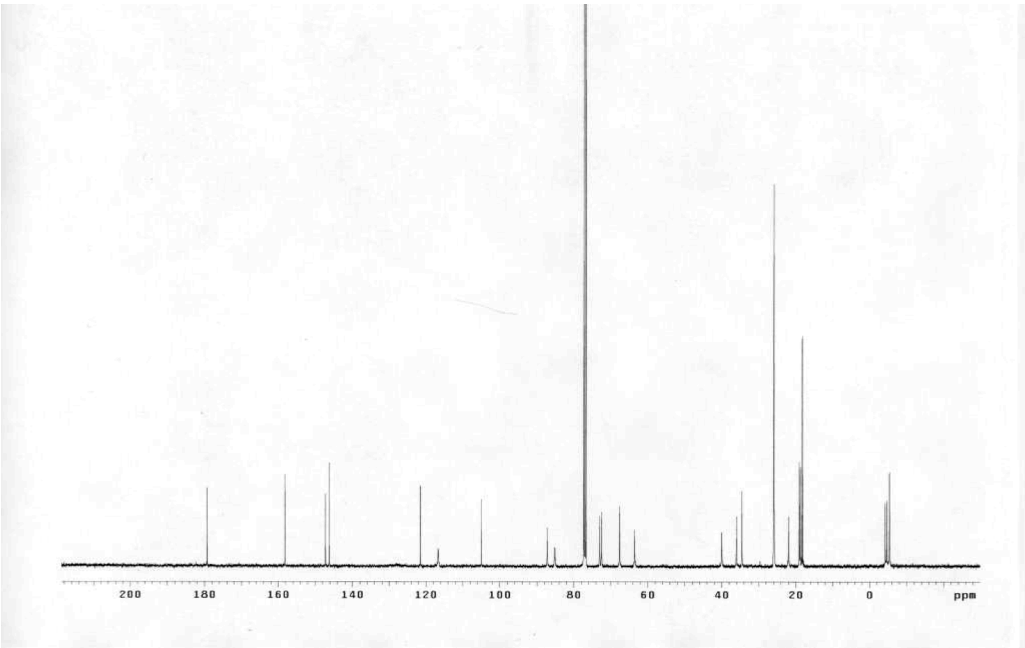

^{1}H & ^{13}C NMR Spectra of 7-deaza-7-(*S*)-(2,3-di-(O-tertbutyl-dimethylsilyl)butyl)-5'-O-(4,4'-dimethoxytrityl)-N(2)-isobutryl-2'-deoxyguanosine

^{1}H

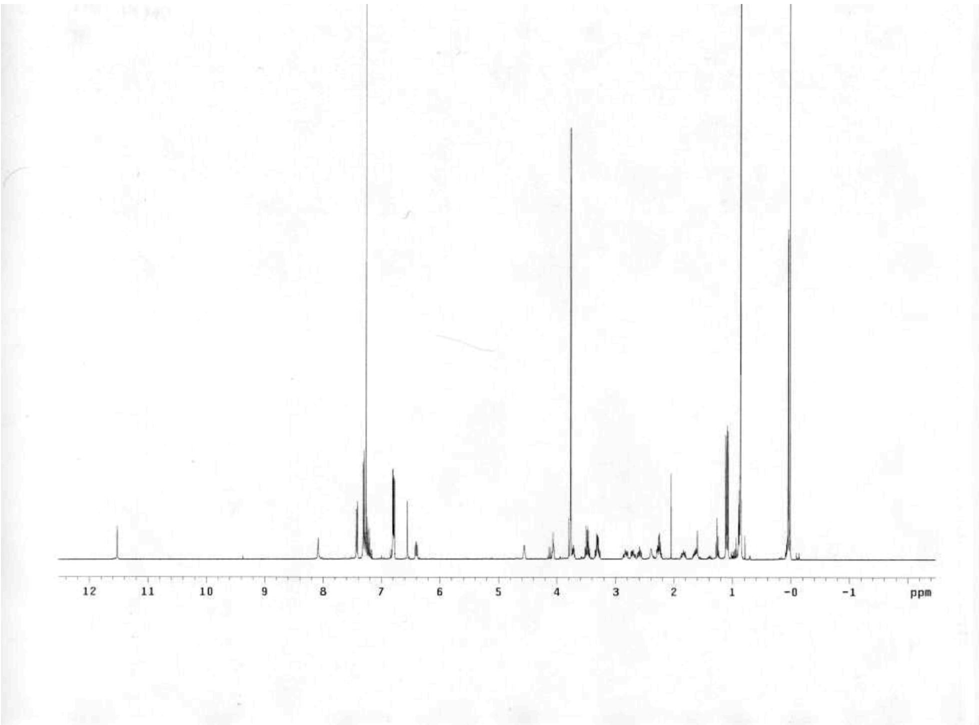

^{13}C

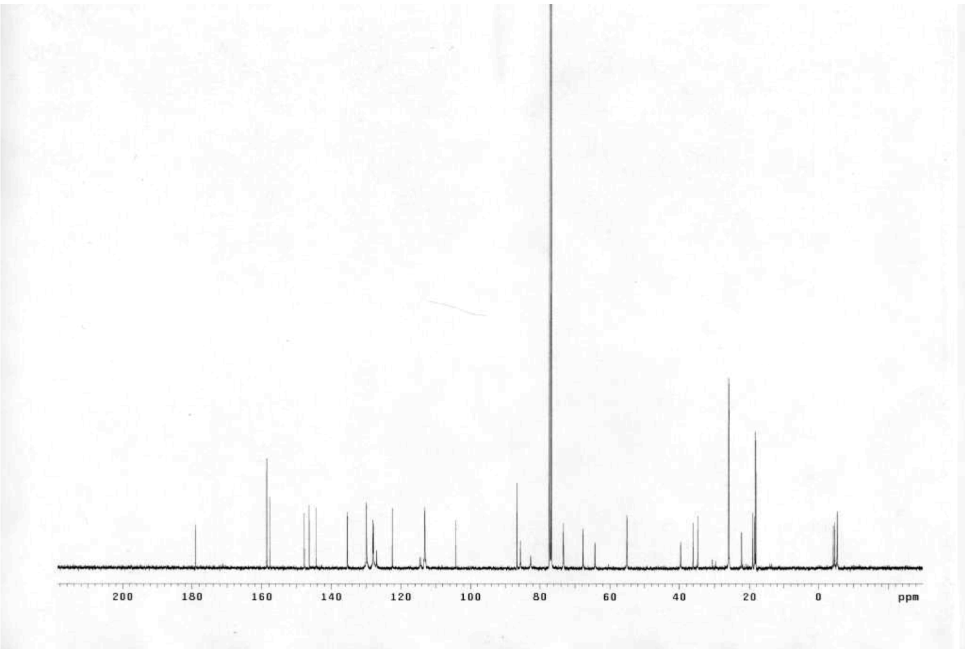

^{1}H & ^{13}C NMR Spectra of 7-deaza-7-(*S*)-(3,4-di-(O-tertbutyl-dimethylsilyl)butyl)-5’-O-(4,4’-dimethoxytrityl)-N(2)-isobutryl-2’-deoxyguanosine-3’-[(2-cyanoethyl)N,N-diisopropylphosphoramidite (8)

[1]H

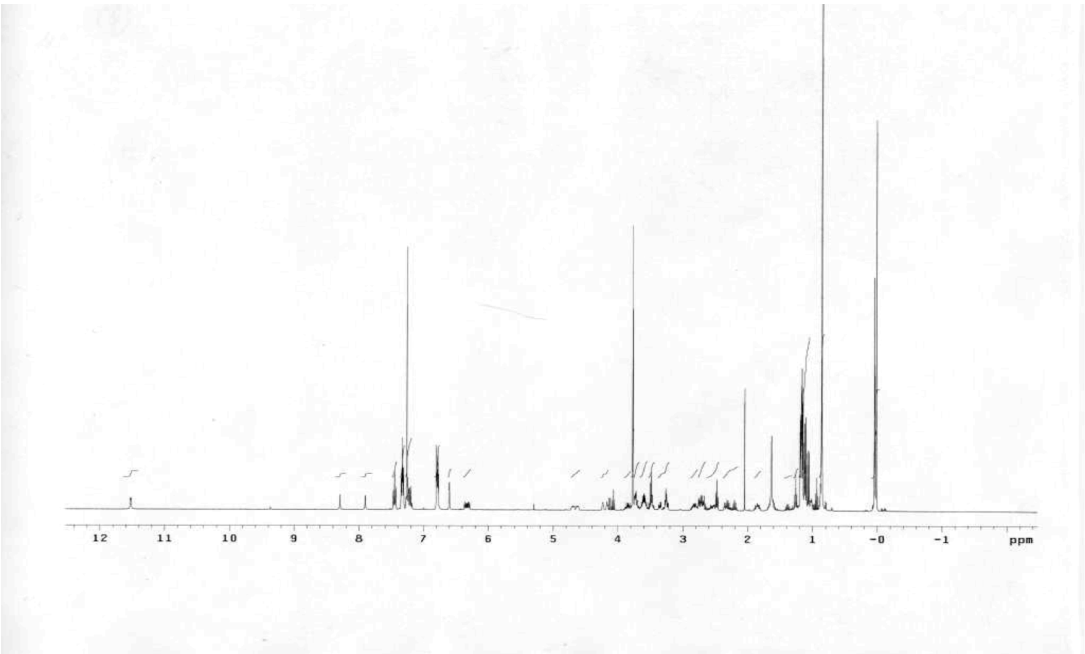

[31]P

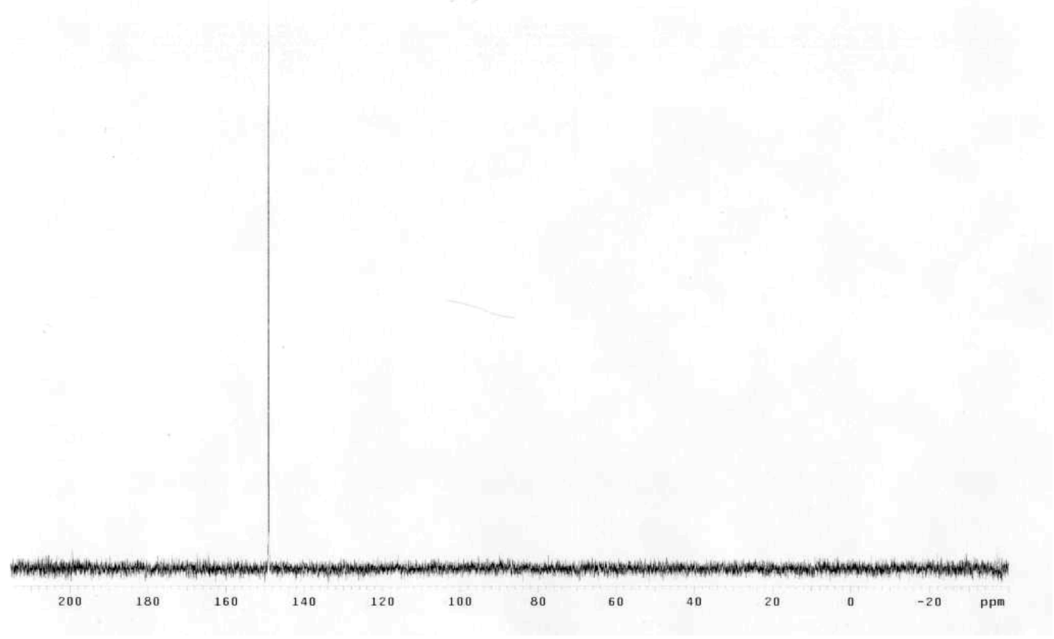

Table 1: MALDI-TOF data of single stranded and ICL oligonucleotides

S1: 5'-GTCACTGGTA**G***ACAGCATTG-3', **S2**: 5'-CAATGCT**G***TCTACCAGTGAC-3'

G*= modified phosphoramidite with either C1/C2/C3 alkyl chain linker

C1, C2, C3: 1, 2 or 3 carbon alkyl chain linker with diol

HY: Hydrazine, DMEDA: N,N'-dimethylethylenediamine

Oligo Sequence	Amine	Calculated m/z	Observed m/z
S1C3	-	6244	6238
S2C3	-	6164	6158
S1C2 x S2C3	NH_3	12315	12331
S1C2 x S2C3	HY	12330	12326
S1C2 x S2C3	DMEDA	12386	12385
S1C3 x S2C3	NH_3	12329	12333
S1C3 x S2C3	HY	12344	12342
S1C3 x S2C3	DMEDA	12400	12391
S1C1 x S2C3	HY	12316	12379
S1C1 x S2C3	DMEDA	12372	12372

MALDI-TOF Images of single stranded and ICL oligonucleotides

MALDI-TOF of ss-oligo S1C3 (5'-GTCACTGGTAGACAGCATTG-3')

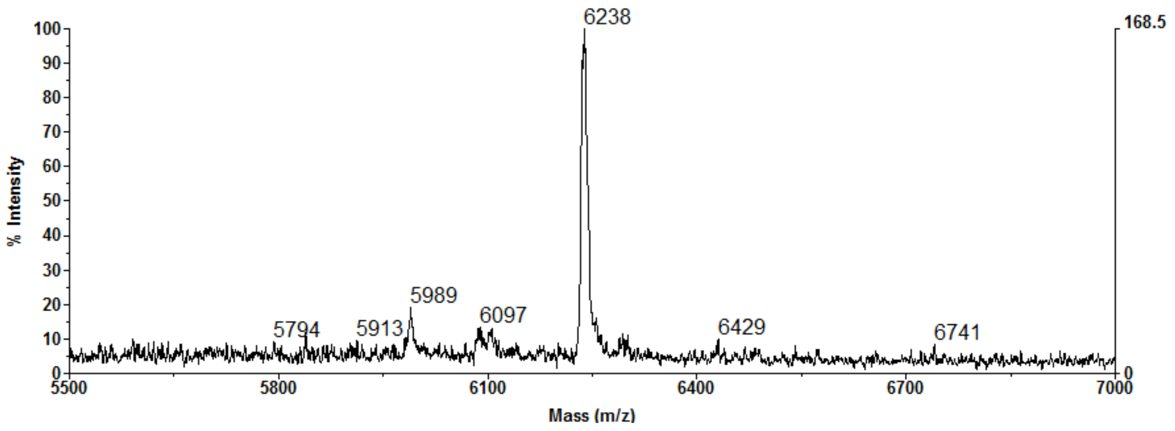

MALDI-TOF of ss-oligo S2C3 (5'-CAATGCTGTCTACCAGTGAC-3')

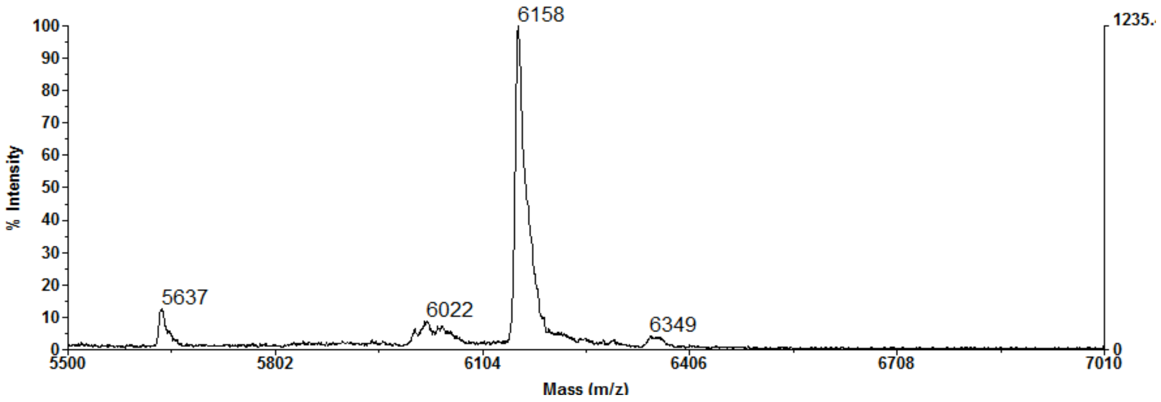

MALDI-TOF of ICL S1C2 x S2C3 + NH_3

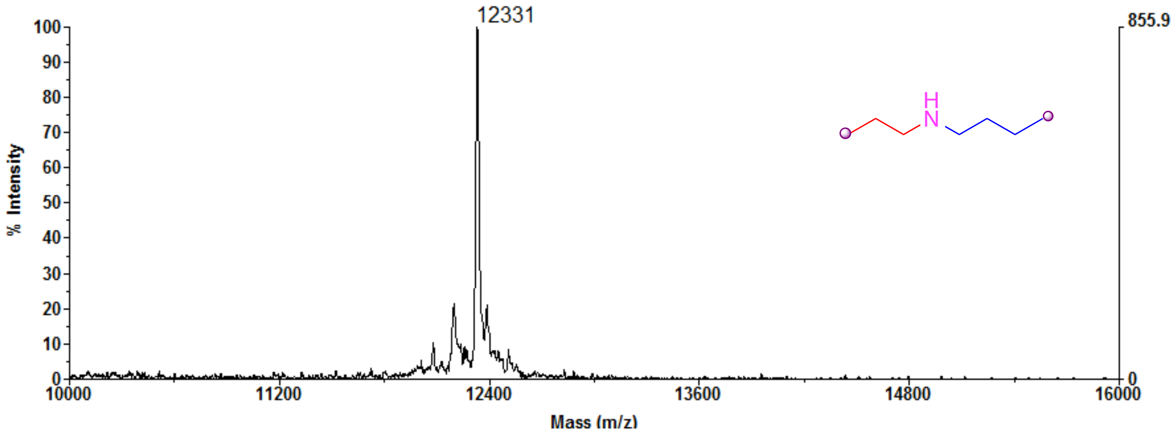

MALDI-TOF of ICL S1C2 x S2C3 + HY

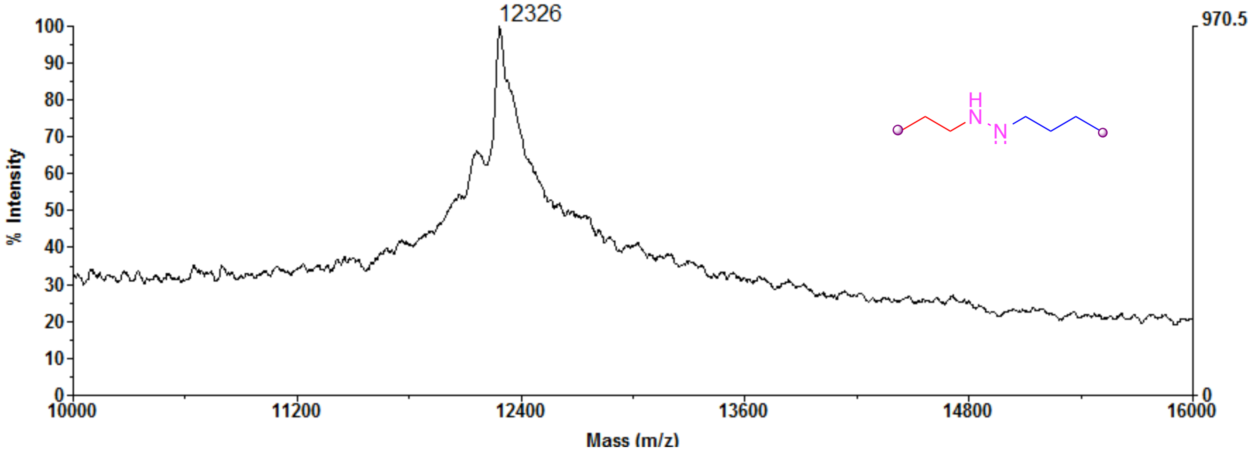

MALDI-TOF of ICL S1C2 x S2C3 + DM

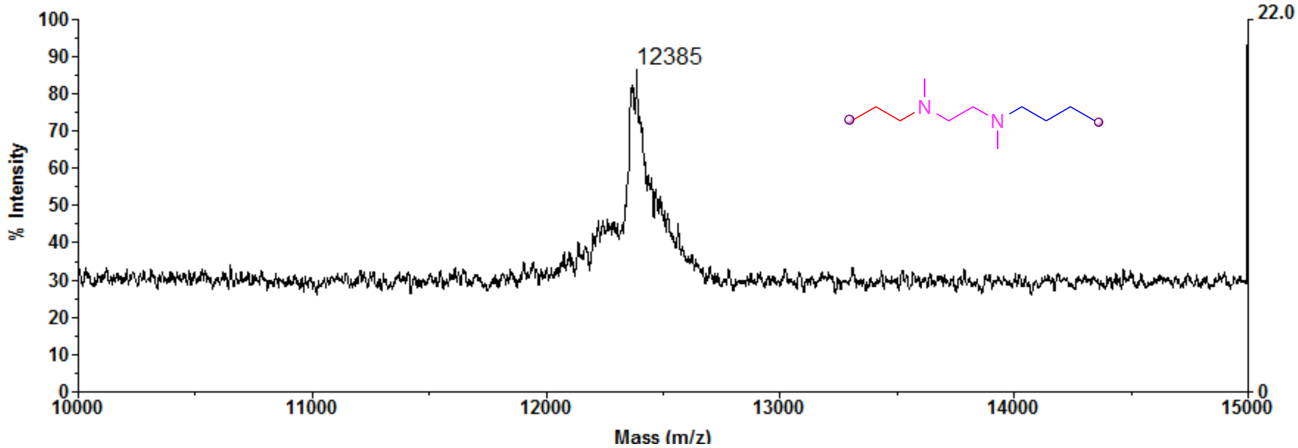

MALDI-TOF of ICL S1C3 x S2C3 + NH_3

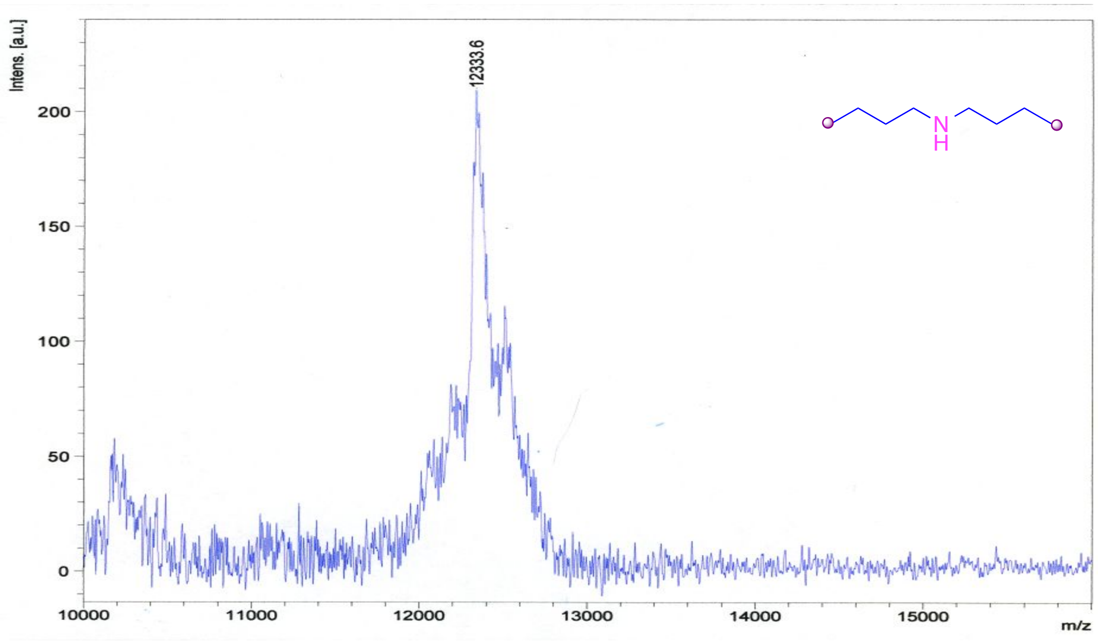

MALDI-TOF of ICL S1C3 x S2C3 + HY

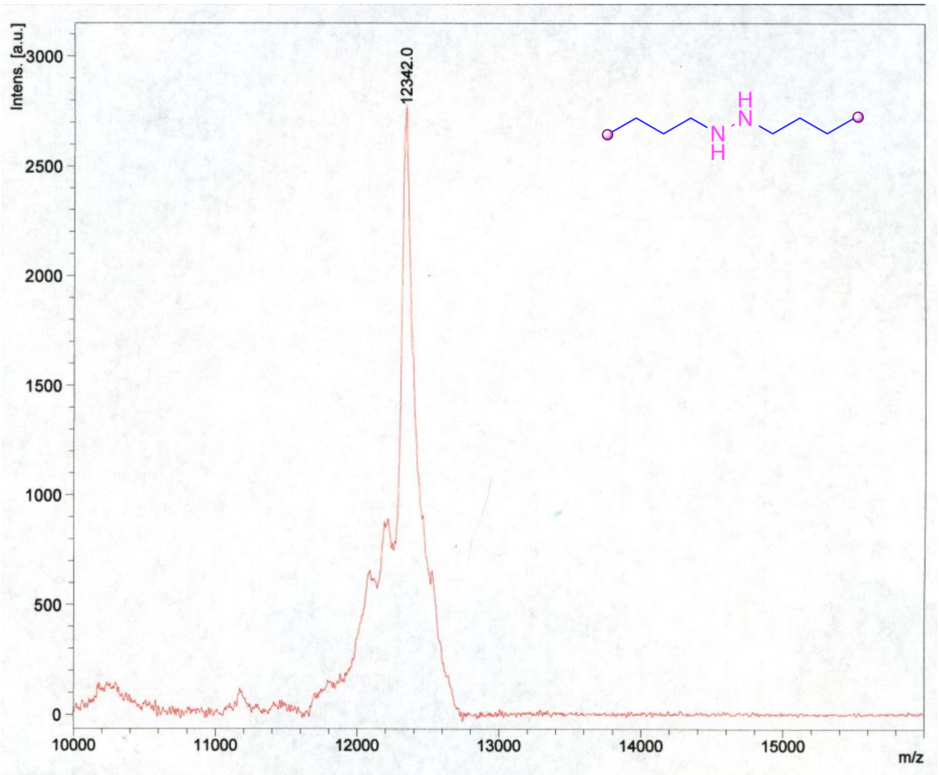

MALDI-TOF of ICL S1C3 x S2C3 + DM

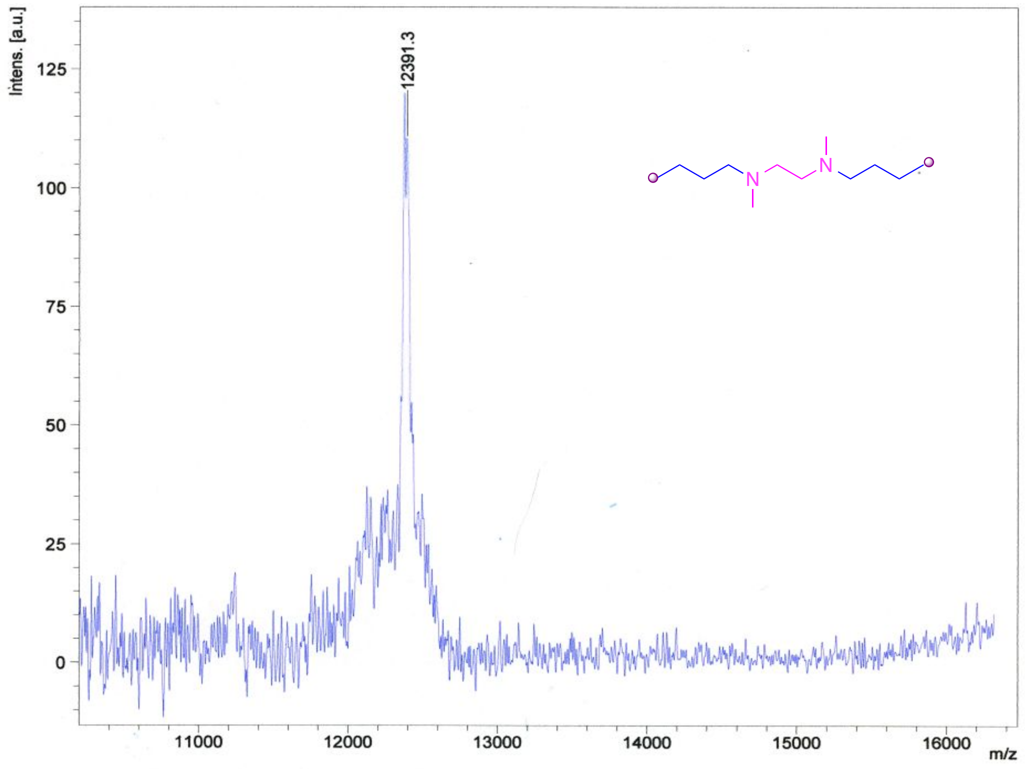

MALDI-TOF of ICL S1C1 x S2C3 + HY

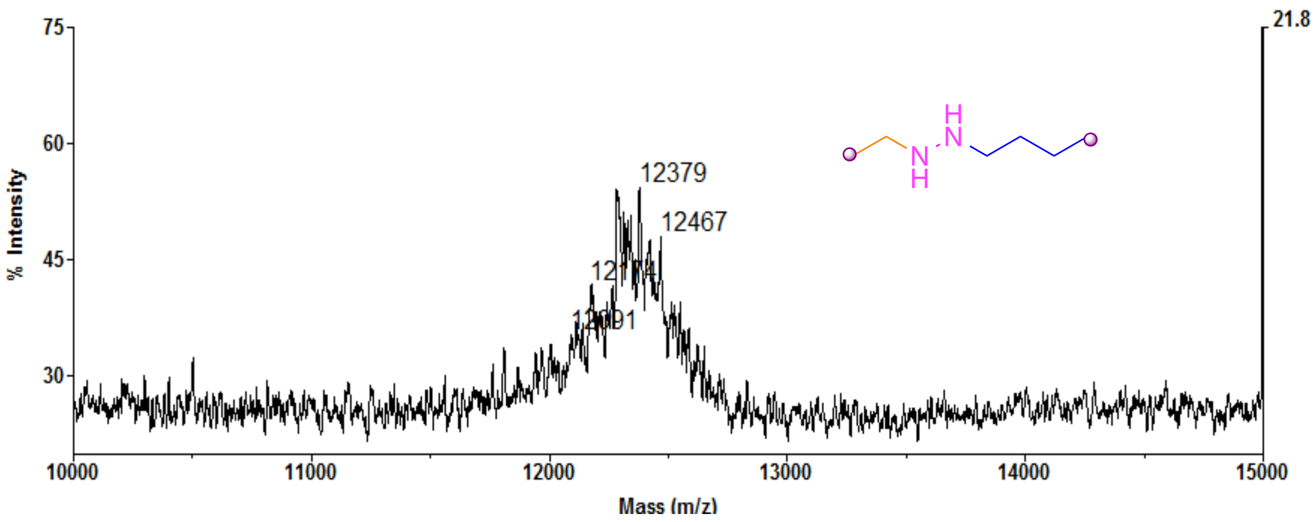

MALDI-TOF of S1C1 x S2C3 + DM

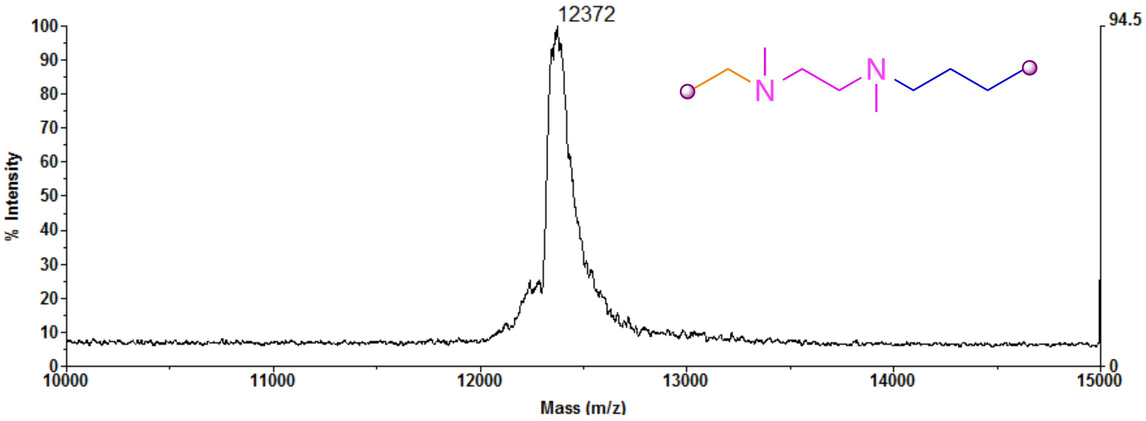

Supplement: SUPPLEMENTARY DATA [file supp_gku328_nar-00601-f-2014-File008.pdf]
